# Shift Work in Nurses: Contribution of Phenotypes and Genotypes to Adaptation

**DOI:** 10.1371/journal.pone.0018395

**Published:** 2011-04-13

**Authors:** Karen L. Gamble, Alison A. Motsinger-Reif, Akiko Hida, Hugo M. Borsetti, Stein V. Servick, Christopher M. Ciarleglio, Sam Robbins, Jennifer Hicks, Krista Carver, Nalo Hamilton, Nancy Wells, Marshall L. Summar, Douglas G. McMahon, Carl Hirschie Johnson

**Affiliations:** 1 Department of Biological Sciences, Vanderbilt University, Nashville, Tennessee, United States of America; 2 Department of Statistics, North Carolina State University, Raleigh, North Carolina, United States of America; 3 Neuroscience Graduate Program, Vanderbilt University, Nashville, Tennessee, United States of America; 4 Vanderbilt School of Nursing, Vanderbilt University, Nashville, Tennessee, United States of America; 5 Children's National Medical Center, Washington, D.C., United States of America; University of Pennsylvania School of Medicine, United States of America

## Abstract

**Background:**

Daily cycles of sleep/wake, hormones, and physiological processes are often misaligned with behavioral patterns during shift work, leading to an increased risk of developing cardiovascular/metabolic/gastrointestinal disorders, some types of cancer, and mental disorders including depression and anxiety. It is unclear how sleep timing, chronotype, and circadian clock gene variation contribute to adaptation to shift work.

**Methods:**

Newly defined sleep strategies, chronotype, and genotype for polymorphisms in circadian clock genes were assessed in 388 hospital day- and night-shift nurses.

**Results:**

Night-shift nurses who used sleep deprivation as a means to switch to and from diurnal sleep on work days (∼25%) were the most poorly adapted to their work schedule. Chronotype also influenced efficacy of adaptation. In addition, polymorphisms in *CLOCK*, *NPAS2, PER2*, and *PER3* were significantly associated with outcomes such as alcohol/caffeine consumption and sleepiness, as well as sleep phase, inertia and duration in both single- and multi-locus models. Many of these results were specific to shift type suggesting an interaction between genotype and environment (in this case, shift work).

**Conclusions:**

Sleep strategy, chronotype, and genotype contribute to the adaptation of the circadian system to an environment that switches frequently and/or irregularly between different schedules of the light-dark cycle and social/workplace time. This study of shift work nurses illustrates how an environmental “stress” to the temporal organization of physiology and metabolism can have behavioral and health-related consequences. Because nurses are a key component of health care, these findings could have important implications for health-care policy.

## Introduction

Sleep/wake patterns, hormone levels, and physiological processes such as core body temperature and heart rate are controlled by a daily biological clock. When behavior and sleep/wake patterns are out of sync with the endogenous clock and/or the environment, such as during shift work, circadian misalignment can result. Misalignment that is a consequence of shift work is associated with an increased risk of developing cardiovascular/metabolic/gastrointestinal disorders, some types of cancer, and mental disorders [1], [2], [3], [4]. In one study, approximately 14% of night shift workers had symptoms that met the criteria for “shift work sleep disorder”, and nearly a third of the shift work sleep disorder workers were depressed. In women, shift workers have a higher incidence of obesity and high blood pressure [5], endometriosis [6] as well as breast cancer [7]. Male rotating shift workers of various occupations as well as male and female shift work nurses are more likely to develop metabolic syndrome (MS) over a 4/5-year period than day-shift controls [8], [9]. Together, these results suggest that identifying factors that contribute to poor sleep timing and sleep quality of night shift workers could be important for ameliorating their health and adaptation to the night shift.

One hypothesis is that there is a discrepancy between environmentally- imposed rest-activity cycles and the endogenous circadian clock in shift workers [10]. Thus, shift workers that have a large discrepancy between their social schedule and their biological time may be awake and working at a time when circadian regulation of core body temperature, mood and performance are at a nadir, thereby contributing to maladaptation and unhealthiness. In rodents, transient internal desynchronization induced by a 6-h delay of the light-dark cycle (1) alters sleep architecture [11] and (2) requires up to six days for clock gene expression rhythms to completely adjust, with different peripheral tissues taking varying amounts of time to shift [12]. The significance of internal desynchronization is underscored by multiple investigations that repeated, weekly 6-h advances in the light-dark cycle in rodents can result in greatly increased mortality in aged or immune-challenged animals [13], [14]. In humans, internal desynchronization can be induced by a forced 28-h sleep-wake cycle (8-h sleep, 20-h awake) which is outside the range of entrainment for the human circadian clock [3]. After four cycles, this protocol results in circadian misalignment, in which the behavioral sleep-wake cycle is 12-h out of phase with the circadian cycle, leptin rhythms are blunted, postprandial glucose and insulin are increased, and cortisol rhythms are 180° out of phase with the behavioral rhythm. Nearly half of the participants undergoing the 28-h cycle exhibited a pre-diabetic state during circadian misalignment. Given that many night-shift workers prefer to sleep at night on their days off (as we found in this study of nurses), internal desynchronization induced by frequent shifts in sleep/wake behavior may contribute to health hazards of shift work.

The timing of the circadian clock is maintained by a set of genes and proteins that form a feedback loop (“clock” genes) as well as rhythmically regulate many output genes (clock -controlled genes). A recent study estimated that nearly 10,000 mammalian genes are rhythmically regulated by clock genes [15]. In addition, polymorphisms of several circadian clock genes have been associated with psychiatric disorders, such as mood disorders and autism [16], [17], [18], [19], [20] as well as metabolic disorders such as metabolic syndrome [21]. Furthermore, circadian clock alleles significantly predict sleep parameters and sleep timing (for review, see [22]). For example, polymorphisms in *PER1* and *PER3* are associated with advanced sleep phase [23], [24], and a rare missense mutation in *PER2* is linked to familial advanced sleep phase syndrome (FASPS) [25]. Finally, a SNP in the *CLOCK* gene predicts delayed sleep timing and reduced sleep duration [26].

Given these links between circadian rhythms and health, we hypothesized that disruptions to the biological clock by shift work have penalties that might be influenced by clock gene polymorphisms. We chose nurses as our subject sample for shift work because (i) their alertness and performance is crucial for health and safety of patients, (ii) they often undergo highly irregular schedules due to attempts to follow a normal day schedule on their days-off for family/social reasons (our nurses' night shift schedule creates a clock stress that is analogous to the jet lag of flying back and forth between Tokyo and San Francisco every few days), and (iii) the adaptation of nurses to night shift work has not been previously reported. We first sought to examine the role of chronotype in the nurses' ability to adapt to night shift work. Second, we compared the effectiveness of sleep strategies commonly used by nurses on both work days and days off. Finally, in an effort to explore gene/environment interactions, we investigated common polymorphisms of circadian or circadian-related genes using both single locus and multi-locus analyses in association with sleep/circadian phenotypes during different shift-work environments. Our results supported our hypothesis of substantial consequences of these highly fragmented schedules to nurses on the night shift.

## Materials and Methods

### Subjects

Participants in the study were nurses who were employees of Vanderbilt University Medical Center (VUMC) and predominantly European American Caucasian (98%). They were a convenience sample recruited through the Vanderbilt University School of Nursing (*n* = 388). Ages ranged from 22 to 76 with a mean, mode, and median of 36.5 (±10.9 SD), 24, and 36, respectively. Most of the nurses were women (*n* = 331 for females, *n* = 52 for males, and *n* = 5 were not indicated). The study was approved by the Vanderbilt University Institutional Review Board (IRB). After obtaining informed consent, participants completed a self-report survey on sleep/wake patterns and had blood drawn for the genetic analyses.

### Phenotyping

The self-report survey was a modified version of the Munich ChronoType Questionnaire (MCTQ; [27]; Figure S1 in Methods S1), in which subjects indicated their current and past shift schedules. The majority worked either 12-h day-shifts (n = 102) or 12-h night-shifts (n = 207; see Table S1 for all shift types). The survey also included a typical schedule for the nurses to indicate 30-min time blocks in which they would normally be sleeping, including any naps. Vanderbilt Hospital night-shift nurses typically work a schedule that includes 3 d on 12-h shifts (7 pm to 7 am) followed by two to five days-off, then on for 3 d, etc. Outcome variables from the survey responses were generated using SPSS 13.0 and are defined as follows.

*Adaptation.* Determined from the survey question that asked: “how well do you feel you adapt to your current work hours?” (see level examples in Figure S1, question #4). Subjects were also asked to respond to additional questions if he/she had previous experience as a hospital shift worker. These questions determined the previous shift schedule, the length of experience with these hours, and adaptation to the previous work schedule. In general, “adaptation” refers to the level indicated in response to question #4 for the current work schedule and/or the previous work schedule. Level “1” referred to feeling tired all of the time, not enjoying days off, and sleep cycles never seem to get regulated. Level “10” referred to having no trouble getting energy back on first days off and sleeping just as well when working as when not working. When adaptation was included in an analysis to determine any association with current behavior (for example, caffeine and alcohol use), only adaptation to the current schedule was used.

*Caffeine and Alcohol consumption.* Determined from open-ended questions asking participants to report the number of caffeinated or alcohol-containing drinks consumed daily (for caffeine) or weekly (for alcohol). *Min out of bed.* Subjects also reported number of minutes needed to get out of bed after waking up on days-off. *Chronotype.* Self-reported on a scale from 0 to 6 (as in the MCTQ).

*Typical schedule variables.* Based on the typical schedule, midsleep time and sleep duration for work days was determined from Day F for night-shifters and from the transition to Day F from Day E for day-shifters (see Figure S1). Midsleep time and sleep duration for free days was determined from Day B for both shift types. Total Sleep Duration for the entire work schedule was also quantified. Additionally, midsleep was adjusted for “sleep debt” (Mid-Sleep Free, Sleep-debt Corrected; MSF_SC_). Sleep debt is defined as midsleep on Free Day B minus sleep debt (0.5*(sleep duration Day B – Total Sleep Duration)/8), as modified from [28]. In order to compare self-reported chronotype with midsleep times, “off-shift sleep phase (corrected for sleep debt)” was generated from the MSF_SC_ variable (as in [27]), where data were grouped into seven 1-h bins, such that the lowest bin referred to midsleep times that were earlier than 2:00AM, and the highest bin referred to times that were later than 7:00AM. A sleep “session” was defined as any sleep period lasting one hour or more. For night-shift schedules, sleep strategies were defined as described in the main text.

### Genotyping

Whole blood samples (5 ml) were collected at VUMC, and DNA was isolated from lymphocytes by the Vanderbilt DNA core facility and used for genotyping by one of three different methods: Sequenom genotyping (Sequenom Inc. San Diego Ca), single-stranded conformation polymorphism (SSCP) analyses [29], [30]], and a third method applied to the variable nucleotide tandem repeat in *PER3* (AB047536), namely to separate the PCR products by electrophoresis in a 2% agarose gel and visualize the difference between the 5-repeat allele (414 bp) and the 4-repeat allele (360 bp) by ethidium bromide staining. Previously reported primers were designed and used for the *ARNTL*, *ARNTL2*, *AA-NAT*, *PER2*, *PER3*, *CLOCK* and *NPAS2* variants as previously described [29]. Details for SNP selection and genotyping procedures for polymorphisms can be found in Methods S1.

### Statistical Analysis

Phenotypes (outcome variables) from the survey responses were analyzed using SPSS version 13.0 for Microsoft Windows (SPSS, Inc., Chicago, IL). The polymorphisms (35 in all, see Table S2) were analyzed for association with the following phenotypes: (i) outcome variables associated with/obtained from the typical schedule chart: work-day sleep duration and midsleep time, free-day sleep duration and midsleep time (unadjusted and adjusted for sleep debt), sleep sessions, off-shift sleep phase (corrected for sleep debt), sleep strategy, total sleep duration, and adaptation, as well as (ii) outcome variables from the remainder of the survey such as alcohol, caffeine, likelihood to doze, self-reported chronotype, and minutes to get out of bed. After covariate selection and careful quality control, polymorphisms were evaluated for potential phenotype associations in both single-locus and multi-loci tests and permutation was used for both regression and GMDR analyses to determine a family-wise type I error rate of 5%, such that raw *p*-values for individual tests of association were considered significant if they were less than 0.009 (see Methods S1). For outcome variables derived from the typical schedule part of the survey, some subjects had completed schedules for both day- and night-shift. To avoid duplicate genetic data for these subjects, the entire dataset was analyzed two ways: first, with all shifts included together (un-stratified) and second, stratified for shift-type during the analysis of these variables. The strength of this stratification approach is that it allowed comparison of associations during different shift environments; however, the weakness of this strategy is that the overall sample size was decreased, resulting in a loss of power. Therefore, it is likely that some associations may not have been detected in our analysis. Moreover, we used the Generalized Multifactor Dimensionality Reduction (GMDR) method [31] to evaluate potential multi-locus interactions that predict each phenotype after adjusting for significant covariates.

## Results

### Behavioral analyses

**Figure 1:**
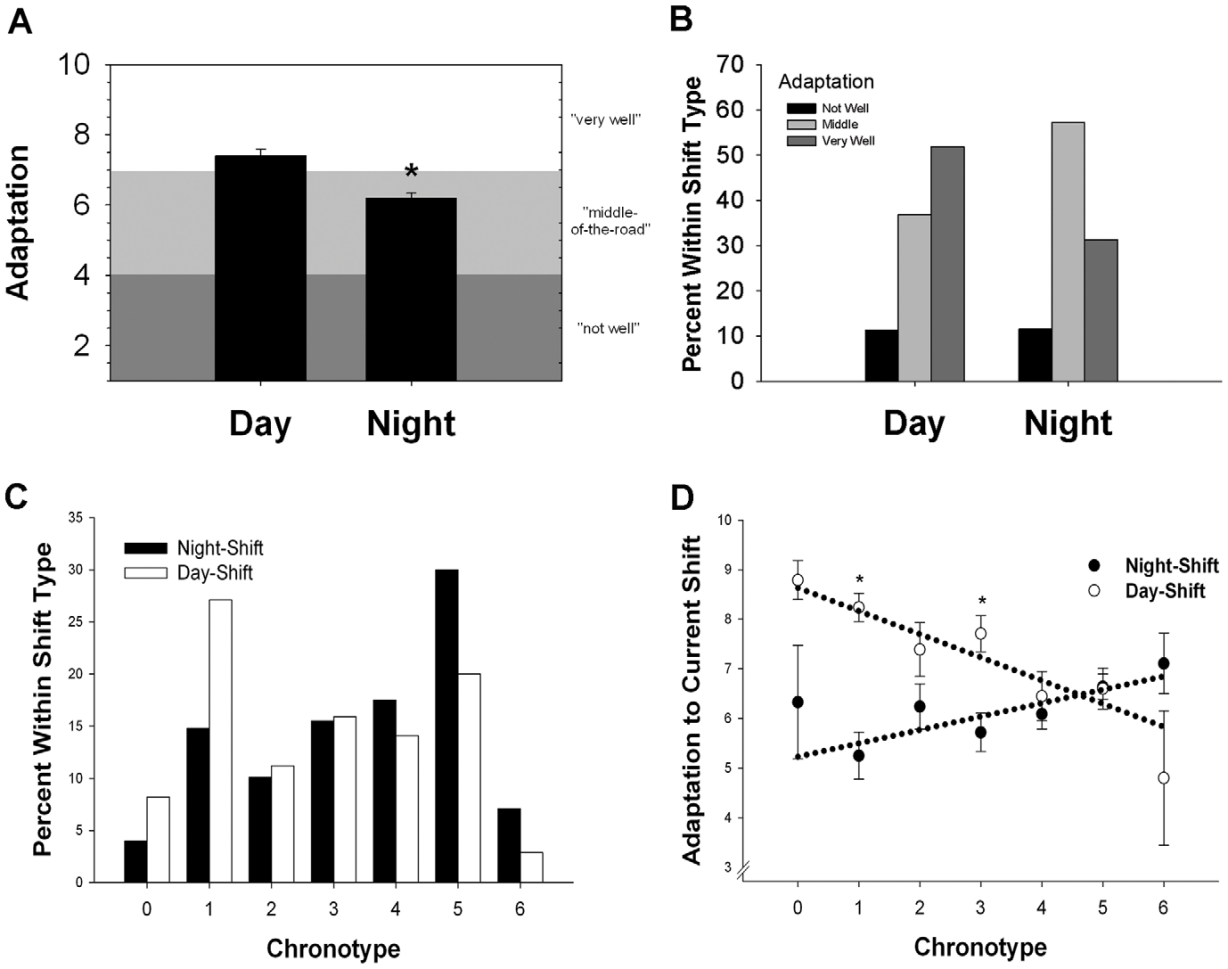
Significant effects of work shift and chronotype on adaptation. **A**) Nurses currently working night-shifts reported significantly lower adaptation levels than those working day-shifts with responses ranging from “1” (feeling tired all of the time, not enjoying days off, and unregulated sleep cycles) to “10” (no trouble getting energy back on first days off and sleeping just as well when working as when not working). Bars represent the mean ± SEM; *t*(360)  = 5.07, *p*<0.01. **B**) A significantly larger percentage of night-shift nurses reported intermediate adaptation levels (corresponding to feeling tired on the first day off and varying sleep patterns) and a smaller percentage reported high adaptation levels compared to day-shift nurses; contingency analysis, G(2)  = 13.6, *p*<0.01. **C**) Histogram of self-reported chronotype for night-shift nurses (black bars) and day-shift nurses (white bars). Night-shift nurses had significantly later chronotypes than did day-shift nurses (*t*(359)  = -4.6, *p*<0.01). **D**) Adaptation to the current shift was significantly predicted by self-reported chronotype for night-shift nurses (black circles) and day-shift nurses (white circles). Circles and error bars represent the mean ± SEM, and dashed line refers to the significant regression equations for each shift type [Night: R^2^ = 0.04, *p*<0.01; Day: R^2^ = 0.13, *p*<0.01]. These data indicate a significant Chronotype X Shift interaction [Scheirer-Ray-Hare extension of the Kruskal Wallis test, H(6)  = 31.0, *p*<0.01].

The majority of the participants worked either 12-h day-shifts (n = 102) or 12-h night-shifts (n = 207; see Table S1 for all shift types). Nurses were asked to rate how well adapted they felt to their current (or past) work schedule, and were given several examples (see Figure S1). Data for shift and age categories (determined by being younger or older than the median age of 36) are summarized in Table 1 for minutes to get out of bed, adaptation to current schedule, caffeine consumption, alcohol consumption, and qualitative (self-reported) chronotype. We first confirmed that night-shift nurses reported significantly lower adaptation to their work schedule than day-shift nurses (*p*<0.01, Figure 1A), and contingency analysis of three categories of adaptation responses revealed that significantly fewer night-shift nurses reported being well-adjusted to their work schedule than did day-shift nurses (*p*<0.01, Figure 1B). Age was significantly correlated with caffeine consumption (Pearson's R = 0.17, *p*<0.01); caffeine consumption did not significantly increase in day-shift nurses over the median age of 36 (G(2)  = 1.6, *p*>0.05) but did significantly increase in night-shift nurses over age 36 (G(2)  = 8.3, *p*<0.05). Night-shift nurses had significantly later chronotypes than did day-shift nurses (*t*(359)  = −4.6, *p*<0.01, see Figure 1C). In order to investigate what effect the self-reported chronotype has on adaptation to shift work, we analyzed the interaction of “Chronotype X Shift” on adaptation levels, and found a significant interaction between these two factors (*p*<0.01, see Figure 1D) such that that earlier chronotypes generally had higher adaptation scores for day-shift and lower ones for night-shift, while later chronotypes had intermediate adaptation levels for both day and night-shifts.

**Table 1:** Nurses responses to survey questions by current shift and median age.

	Night-shift				Day-shift			
	≤36		>36		≤36		>36	
	Mean	N	Mean	N	Mean	N	Mean	N
Min Out of Bed	13.9±1.0	118	12.1±1.1	81	11.9±1.1	63	10.8±1.4	71
Adaptation	6.1±0.2	121	6.2±0.3	85	7.7±0.2	68	7.2±0.3	83
Caffeine (Drinks/Day)	2.6±0.2	121	3.5±0.2	85	2.5±0.2	68	3.4±0.4	83
Alcohol (Drinks/Wk)	1.4±0.2	118	1.2±0.3	83	1.7±0.3	67	1.2±0.2	82
Chronotype (self-reported)	3.7±0.1	121	3.5±0.2	85	3.0±0.2	69	2.7±0.2	84

**Figure 2:**
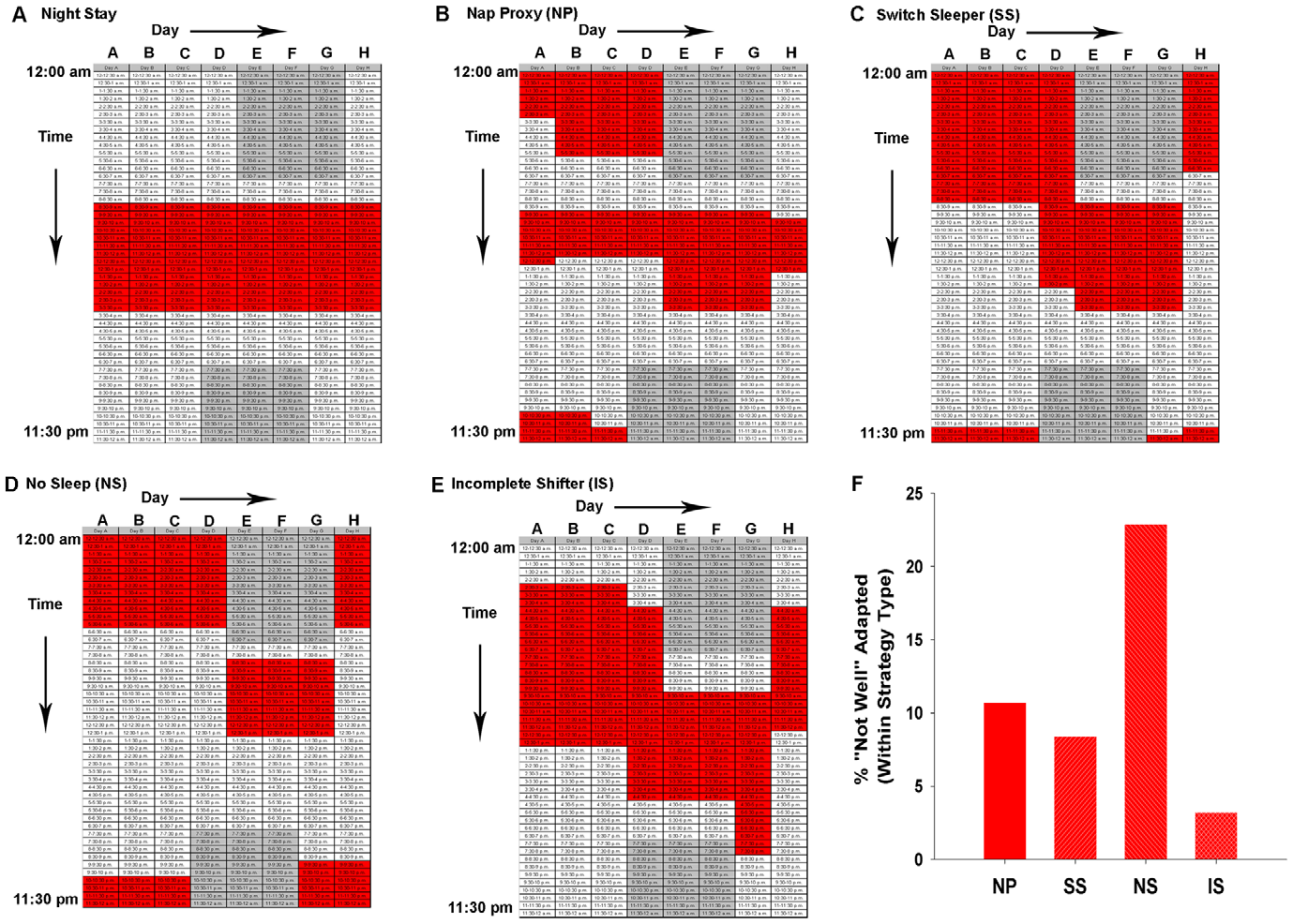
Representative sleep schedules for typical night-shift schedules. Nurses were instructed to shade the time boxes for any time in which they would sleep, including any naps for only the schedules they had actually experienced. Some nurses had experienced both night-shift or day-shift and they completed both work-week schedules, but most nurses completed only one of the schedules. Each column is one 24-h period beginning at 12:00am, and each box represents 30-min. Gray shaded area refers to the night-shift work schedule (7:00pm to 7:00am for the typical night-shift schedule at Vanderbilt Hospital). Red shaded areas represent typical responses for sleep time in the surveys. These responses were categorized into five strategy types: **A) Night Stay:** Continued to sleep regularly in the daytime on or off shift; **B) Nap Proxy (NP):** On days off, they nap (longer than one hour) on at least four out of the five days off during the time in which they would normally be asleep when working night-shift; **C) Switch Sleepers (SS):** Switch from nights to days by using a strictly enforced schedule, but they do not give up any sleep in order to do so (i.e., they sleep late on the day they will start night-shift work); **D) No Sleep (NS):** Switch from days to nights and vice versa by choosing a>24-hr period to stay awake entirely; **E) Incomplete Switcher (IS):** Switched half-way between days and nights; **F)** Post hoc analysis of significant MANOVA for strategy type [F(12, 558)  = 1.8, Pillai's Trace = 0.11, p<0.05] of nurses who were currently working night shift and did not choose the Night Stay strategy. Significant univariate analysis revealed a significantly greater percentage of nurses (within Strategy Type) falling into the “Not Well” Adapted category (G(3)  = 8.7, *p*<0.05). Frequencies of nurses who reported being “Not Well” Adapted (for each group from left to right): 3/27, 8/92, 10/46, and 1/31.

We next determined how adaptation translates to sleep phase, duration, and the frequency/timing of sleep sessions, by including two 8 d work schedules in the survey that are typical for shifts at Vanderbilt Hospital (and many other hospitals) — one for day shifts and one for night shifts. Nurses were instructed to shade the hourly boxes in order to indicate the typical times in which they would sleep and/or nap when working that particular schedule. (Sample responses are represented in Figure 2A–E). Self-reported sleep times accurately reflect and are significantly correlated with ambulatory monitoring such as actigraphy [27], [32], [33]. We determined the times of midsleep for a typical work day (Day F) and free day (Day B) after adjusting for sleep debt ( =  sleep deprivation that accumulates over time; calculated as in [28], see Methods). There were significant main effects of Age and Shift (*p*<0.05, Table 2), such that midsleep times were significantly earlier for older nurses and day-shift nurses; however, there was no significant Age X Shift interaction (*p*>0.05, Table 2). Despite these phase differences, shift type did not affect the duration of sleep on work days, except for nurses over the median age of 36, where older night-shift nurses had significantly shorter sleep durations on work days than younger night-shift nurses (*p*<0.01, Table 2). This age dependency for sleep duration was not evident in day-shift workers. In addition, results of a two-way analysis of variance (ANOVA) revealed that shift type did not significantly affect sleep duration on Free Day B nor on total sleep duration for the entire 8-day cycle (main effect of shift-type: F(1,457)  = 0.03, *p*>0.05 for Free Day B and F(1,455)  = 0.00, *p*>0.05 for total sleep duration, Table 2) despite a significant increase in the number of indicated sleep sessions (including naps) throughout the 8-d work-cycle for night-shift nurses as compared to day-shift nurses (*p*<0.01, Table 2). For sleep duration on free days and the total work week, there was a significant main effect of age (*p*<0.01) but no significant interactions of shift type with age (*p*>0.05, Table 2).

**Table 2:** Sleep phase, duration, and the frequency/timing of sleep sessions from typical shift schedules.

	Night-shift				Day-shift			
	≤36		>36		≤36		>36	
	Mean	N	Mean	N	Mean	N	Mean	N
Midsleep Time (Work)	12:13±:03	156	12:16±:05	133	01:33±:04	76	01:28±:04	92
Midsleep Time (Free) Adj	04:22±:11	156	03:19±:12	133**,†	02:51±:07	76	02:19±:05	92
Duration (h) - Freeday B	9.48±0.14	156	8.82±0.15	133**	9.38±0.15	76	8.82±0.16	92
Duration (h) - Workday E	7.46±0.12	156	6.80±0.13	133*	7.28±0.11	76	7.08±0.10	92
Sleep Sessions (#/week)	9.23±0.13	156	9.36±0.18	133†	8.64±0.17	76	8.55±0.14	92
Total Duration (h/week)	70.15±0.75	156	64.32±0.92	133**	69.51±0.90	76	65.50±0.98	92
Adaptation	5.95±0.18	132	5.96±0.27	106	7.73±0.27	60	7.45±0.29	65

Values represent mean ± SEM for two age groups (≤36 and>36). Adj – adjusted for sleep debt (see Methods).

*Significantly different from younger night-shift nurses, Kruskal Wallis χ^2^(3)  = 20.5, Tamhane's posthoc, *p*<0.01.

**Significant main effect of age: adjusted midsleep on freedays, Scheirer-Ray-Hare extension of the Kruskal Wallis test, H(1)  = 33.0, *p*<0.01; sleep duration on freedays, ANOVA, F(1,457)  = 7.9, *p*<0.01; and Total Duration, ANOVA, F(1,455)  = 16.4, *p*<0.01.

† Significantly different from day-shift nurses, regardless of age: adjusted midsleep on freedays, Scheirer-Ray-Hare extension of the Kruskal Wallis test, H(1)  = 45.4, *p*<0.01; Work offset – Sleep onset difference, ANOVA, F(1,452)  = 272.5, *p*<0.01; Sleep sessions, Mann-Whitney U = 18,110, *p*<0.01.

### Sleep “strategies” predict adaptation to nurse night shift schedules

The observation that sleep duration is the same between night-shift and day-shift nurses, but that adaptation is poorer for night-shift nurses implies that the phasing of sleep relative to the underlying (and disrupted) circadian oscillator is important for optimal adaptation. Therefore, it may be that adaptation to night shift is affected by not only how much sleep the nurses get, but when they sleep relative to their work episode. Several studies have indicated that the sleep/wake patterns of simulated shift workers on days-off can significantly affect night-shift performance [34], [35], [36], [37]. Here, we distinguished five different sleep strategies that the Vanderbilt Hospital nurses chose for days-off (represented in Figure 2A–E). Some nurses chose to stay on night-shift throughout the week (“Night Stay,” Figure 2A). Three strategies distinguished those who switched completely from nights to days on days-off. The “Nap Proxy” strategy was used by those nurses who indicated that they typically nap (longer than one hour) nearly every day during the time in which they would normally be asleep when working night-shift (Figure 2B). “Switch Sleepers” switched from nights to days by using a strictly enforced schedule, but they did not sleep deprive themselves in order to do so (Figure 2C). In contrast, the “No Sleep” strategy was followed by nurses that switched between day- and night-shifts by choosing a >24-h period to stay entirely awake (thereby voluntarily choosing to deprive themselves of sleep, Figure 2D). A final group switched half way between days and nights rather than switch entirely to day-shift on days-off (“Incomplete Switcher,” Figure 2E).

**Figure 3:**
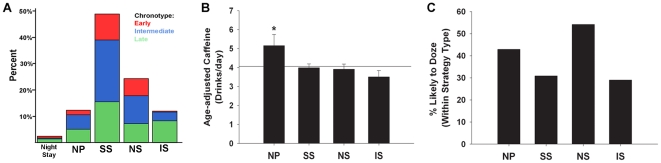
Post hoc analyses of significant MANOVA for strategy type. **A)** Chronotype distribution for Strategy subtypes: Bars represent the response rate for each strategy type and the colors represent chronotype, where the colored areas within each bar correspond to the percentages of nurses with that chronotype within the strategy type (Total N = 295). Red = early (scoring 1–2); blue = intermediate (scoring 3–4); green = late (scoring 5–6). **Panels B,C:** Univariate analysis of nurses who were currently working night shift and did not choose the Night Stay strategy (the Night Stay group was excluded due to its small sample size) revealed significant effects of age-adjusted Caffeine consumption **(panel B;** contrast comparisons against the mean, *p*<0.01) and percentage (within Strategy Type) falling into the moderate to high likelihood to doze category **(panel C;** G(3)  = 8.6, *p*<0.05). See also Table 2 for sleep strategy descriptives.

The response rate and chronotype distribution for each strategy is depicted in Figure 3A. The most common strategy was the Switch Sleeper strategy (∼50%), with the second most common strategy being No Sleep (∼25%). Previous reports did not prepare us for the observation that fully 1 out of 4 night-shift nurses chose to switch between days and nights via a >24-h sleep deprivation period (the No Sleep strategy). In our sample, the sleep deprivation commonly occurred just before the first work day, which could impair performance and alertness on the job. While strategy types did not differ with regard to sex or schedule rotation, the strategy types did differ in terms of nurses' age, years of experience, children in the home, and chronotype (see Table 3 for descriptive and inferential statistics). Generally, Incomplete Switchers had the latest chronotypes, while the Sleep Switchers and No Sleep nurses had significantly earlier chronotypes compared to the Incomplete Switchers (see Figure 3, panel A). In addition, older and more experienced nurses were more likely to choose the No Sleep strategy or Night Stay strategies, while nurses with children at home were less likely to be an Incomplete switcher and more likely to choose the No Sleep strategy.

**Table 3:** Descriptive Statistics for Strategy Types.

	Night Stay	Nap Proxy	Switch Sleepers	No Sleep		Incomplete Switcher
Age	39.1±3.9	34.3±1.4	35.2±0.9	40.5±1.3	32.8±2.0	
Years of Shift Experience	3.2±0.2	2.4±0.3	2.7±0.1	3.3±0.2	2.6±0.3	
Number of Children in Home	0.3±0.7	0.6±0.1	0.4±0.0	0.6±0.1	0.2±0.1	
Chronotype	3.9±0.8	3.7±0.3	3.3±0.1	3.1±1.2	4.5±0.2	

Notes: Numbers are means ± SEM. Strategy Type significantly predicted self-reported chronotype, such that Incomplete Switchers had the latest chronotypes, while the Sleep Switchers and No Sleep nurses had significantly earlier chronotypes compared to the Incomplete Switchers (Kruskal-Wallis, χ^2^(4)  = 20.9, *p*<0.01, Tamhane's *post hoc* comparisons, *p*<0.01). Strategy Type significantly predicted age with older and more experienced nurses more likely to choose the No Sleep strategy or Night Stay strategies (ANOVA, F(4,286)  = 4.5, *p*<0.01; Tukey's HSD, *p*<0.05). There was a significant effect of Strategy Type on Years of Experience (ANOVA, F(4,226)  = 2.4, *p*<0.05), such that experienced nurses were more likely to choose the No Sleep strategy than the Nap Proxy, No Sleep, or Incomplete Switcher strategies (*p*<0.05). Nurses with children at home were less likely to be an Incomplete switcher and more likely to choose the No Sleep strategy (G(4)  = 21.0, *p*<0.05). Nearly a third (29%) of nurses with children in the home chose No Sleep compared to one-fifth of the nurses without children in the home. Only 4% of nurses with children in the home were Incomplete Switchers compared to 18.2% of nurses without children in the home.

In order to quantify which strategy was associated with the best adaptation for shift work, we selected the nurses who were currently working night-shift and performed a multivariate analysis of variance (MANOVA) using multiple variables that could be indicative of adaptation. We also excluded the Night Stay strategy due to the low frequency of this strategy type (N = 7/295). We found strategy-specific effects on the “likelihood to doze”, the number of caffeine beverages consumed/day, and self-reported adaptation level (MANOVA, *p*<0.05). Further univariate *post hoc* analysis revealed that nurses adopting the No Sleep strategy resulted in significantly lower adaptation levels (5.58±0.39 compared to 6.11±0.16, *p*<0.05) – nearly 25% of No Sleepers reported being “not well adapted” compared to ∼3–10% of the nurses choosing one of the other switching strategies (*p*<0.05; see Figure 2F). Moreover, the Nap Proxy nurses consumed significantly more caffeine (*p*<0.01; Figure 3, panel B) and were more likely to doze during sedentary activity along with No Sleep nurses (*p*<0.05; see Figure 3, panel C). There were no significant strategy-specific effects on the number of minutes to get out of bed (*p*>0.05).

### Gene/Environment Interactions: Circadian genotype/phenotype associations depend on shift-work environment

In addition to the social factors described above, we investigated whether the environment (of shift work) could combine with genetic variation to potentially influence the adaptation of nurses to their schedules (for review, see [38]). The specific clock gene variants studied here were previously identified to be common polymorphisms in clock genes using a candidate gene approach [29](see Methods S1). While the overall contribution of rare versus common polymorphisms to trait variation is unknown for the traits of interest in the current study, a candidate gene approach looking for predictive common variation was performed based on the key advantages of this tactic such as high power, ability to generalize, and previous successes of such an approach.

#### Single-locus associations

**Figure 4:**
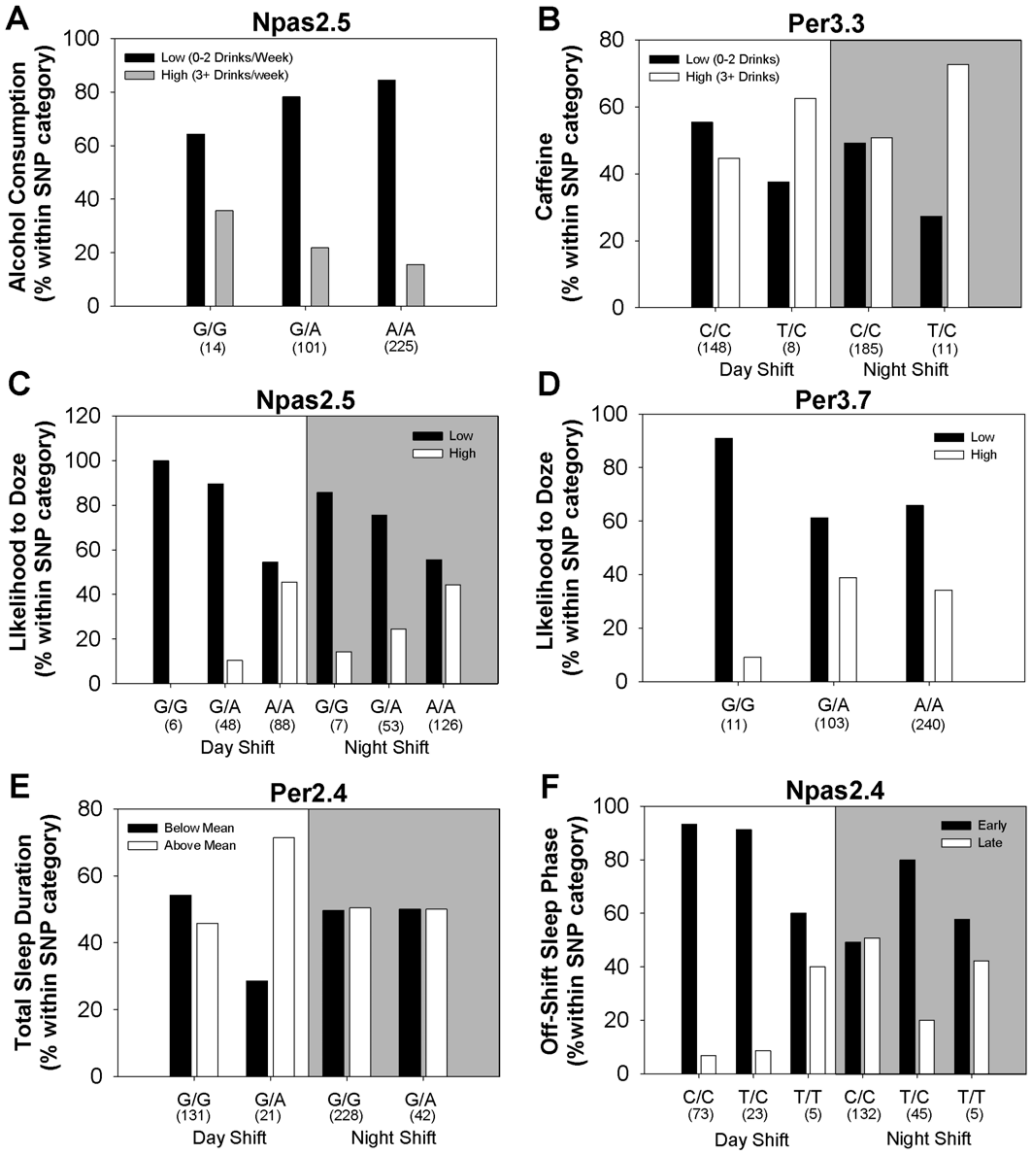
Circadian clock gene SNPs significantly associated with phenotypes. (**A–D**) Significant single-locus associations with alcohol and caffeine consumption and likelihood to doze (*p*<0.009) were identified via regression analysis. The following contingency plots graphically depict these associations: (**A**) an intronic SNP (NPAS2.5) in *NPAS2* predicted alcohol consumption independent of current shift type, R^2^ = 0.08, *p*<0.009. (**B**) A SNP in exon 17 (PER3.3) of *PER3* predicted caffeine consumption for both day- and night-shifters (covariates: age and children at home, R^2^ = 0.15, *p*<0.009). (**C**) An intronic SNP (NPAS2.5) in *NPAS2* predicted the likelihood to doze for both shift types, R^2^ = 0.08, *p*<0.009. “Low” refers to zero or slight chance of dozing and “High” refers to a moderate or high chance of dozing (see Question 10 in Figure S1); (**D**) the PER3.7 SNP predicted likelihood to doze independent of current shift type, R^2^ = 0.10, *p*<0.009. (**E**,**F**) Two gene SNPs were significantly associated with variables obtained from the typical schedule (*p*<0.009). (**E**) Using age and children at home as covariates, total duration of sleep for the work-cycle was significantly associated with PER2.4 (covariates: age and children at home, R^2^ = 0.09, *p*<0.009). Graph depicts the percentage of nurses within each genotype with sleep durations that fell above or below the mean for all nurses (67.3). This association was only significant for day-shift nurses. (**F**) Off-shift sleep phase (corrected for sleep debt as in [10]) was significantly associated with an NPAS2.4, R^2^ = 0.09, *p*<0.009. Graph depicts the percentage of day- and night-shift nurses (within each genotype) that had off-shift sleep phases that were early (lower 33%, with a mid-sleep time<3.0) or late (upper 33%, with a mid-sleep time>4.0). All panels: numbers in parentheses below each bar indicate the number of cases with that genotype.

Significant single locus associations with phenotypes were identified within the *NPAS2* and *PER3* genes. First, alcohol consumption was significantly associated with the NPAS2.5 SNP. Regardless of current shift type, nurses with the *GG* genotype for this SNP had higher average weekly alcohol intake than those carrying *AG* or *AA* (corrected *p*<0.05; Figure 4A). Second, a nonsynonymous, exonic SNP within the *PER3* gene (PER3.1) significantly predicted caffeine consumption for both day- and night-shifts, with *TC* heterozygotes reporting higher daily caffeine use compared to *CC* homozygotes (corrected *p*<0.05; Figure 4B). Interestingly, two SNPs were associated with a decreased likelihood to doze. Specifically, *GG* homozygotes for the NPAS2.5 SNP in *NPAS2* and *GG* homozygotes for the PER3.7 exonic SNP in *PER3* were more likely to report slight or no chance of dozing during sedentary activity, even for nurses working night-shift (corrected *p*<0.05, Figure 4C,D). However, these two SNPs did not synergistically interact to predict likelihood to doze (see Table 3).

#### Gene-Environment interactions

The significant single locus, genotype-phenotype associations presented thus far were detected from the un-stratified dataset and did not take into account shift-type. However, it is clear that environment not only contributes to but also interacts with genetics to influence behavior (for review, see [39]). In the case of this study, day- vs. night-shift is effectively two alternative environments to which for the biological clock attempts to entrain, and we therefore sought to identify any gene-by-environment interactions by stratifying the data-set by shift-type. Two SNPs, one in *PER2* and one in *NPAS2,* were significantly associated with variables obtained from the typical schedule and were specific to either day-shift or night-shift. For example, the total duration of sleep for the entire schedule was more likely to be above the mean for *GA* heterozygote nurses on day-shift than *GG* homozygotes at the PER2.4 locus of *PER2* (*p*<0.009, Figure 4E), but not for night-shift nurses. The NPAS2.4 SNP was associated with off-shift sleep phase (corrected for sleep debt; as in [28], see Methods). In general, sleep phase on free days was earlier for day-shift and later for night-shift (Table 2). However, day-shift nurses carrying *TT* for the NPAS2.4 SNP were more likely to have a later off-shift sleep phase (corrected for sleep debt) than those carrying *CT* or *CC* (*p*<0.009, Figure 4F). For *CT or CC* genotypes, the percentage of late chronotype nurses was higher for night-shift than for day-shift, but the percentage of late-chronotype *TT* homozygotes did not increase on night-shift compared to day-shift.

**Figure 5:**
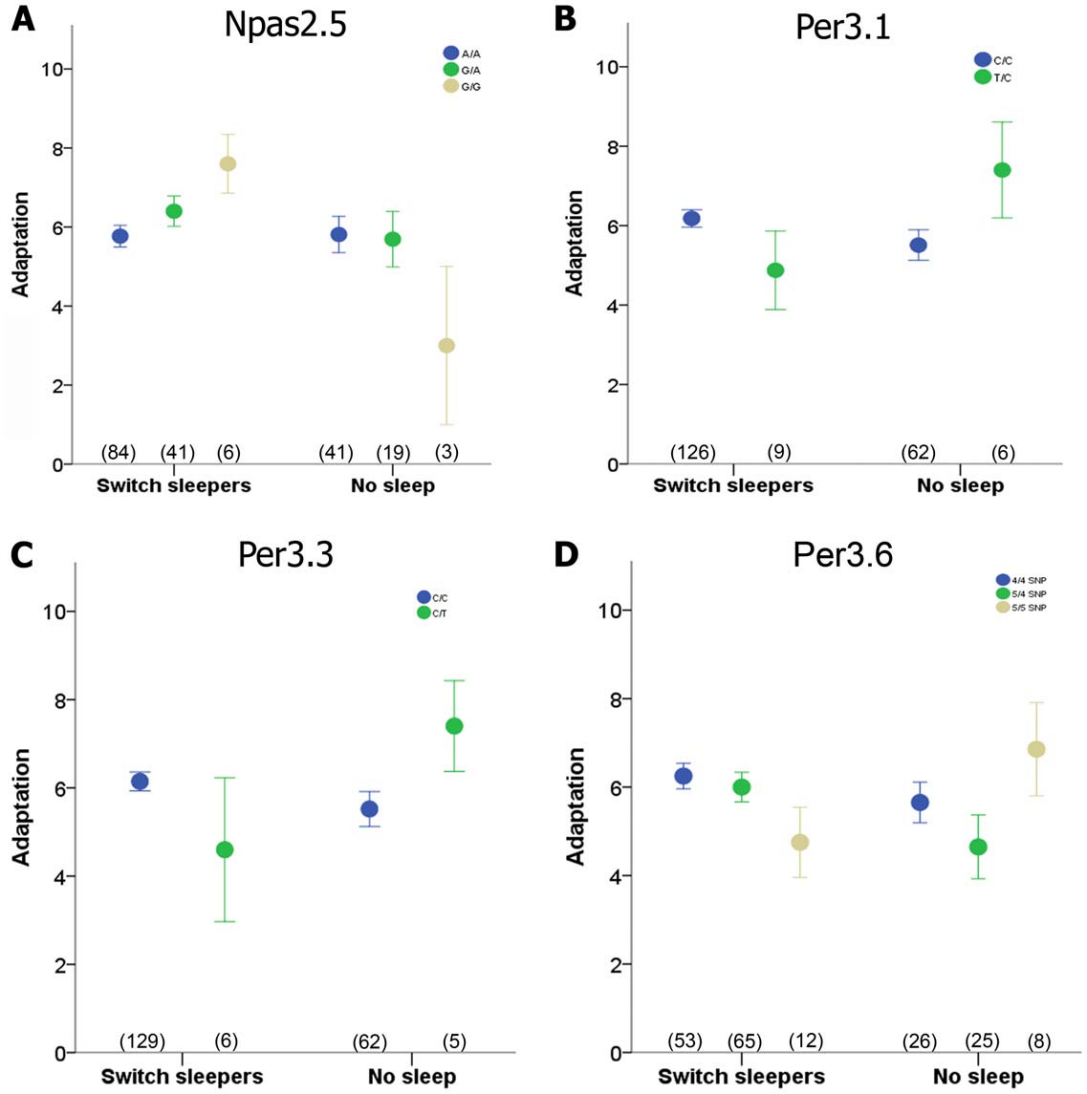
The effect of strategy on self-reported adaptation depends on genotype. Significant Strategy X Genotype interactions for the two most common strategies (Switch Sleepers and No Sleep) were identified by two-way ANOVA. Significant results were not corrected for multiple testing of polymorphisms due to the low sample size. Polymorphisms with significant interactions and corresponding *F* statistics were: (A) NPAS2.5 in *NPAS2,* F(2,151)  = 3.4, *p* = 0.04; (B) PER3.1 in *PER3*, F(1,157)  = 5.0, *p* = 0.03; (C) PER3.3 in *PER3,* F(1,156)  = 4.7, *p* = 0.03; (D) and PER3.6 in *PER3*, F(2,146)  = 3.2, *p* = 0.04 (D). Circles and error bars represent the mean and SEM, and sample size for each genotype is listed under the corresponding circle.

For some genotypes, certain sleep strategies were more or less beneficial based on self-reported adaptation levels. Here, our results provide suggestive evidence that some genotypes adapt more poorly when sleep deprivation is used to switch from days to nights and vice versa, but that this same strategy enhances adaptation for other genotypes. Specifically, we examined adaptation levels of the two most common strategies (Switch sleepers and No Sleep) and found significant Genotype X Strategy interactions for four polymorphisms (NPAS2.5, PER3.1, PER3.3, and PER3.6) using univariate analyses (two-way ANOVA; see Figure 5). Thus, genotype is an important consideration when shift workers adopt a strategy for adjustment to the night-shift schedule.

#### Multi-locus associations

In addition to considering the influence of environment (including social/workplace factors) on clock-related phenotypes in our nurse population, we also set out to test whether multi-locus genetic effects (either intra- or inter-genic) will interact to influence the behaviors we studied herein. We used the Generalized Multifactor Dimensionality Reduction (GMDR) method [31] to evaluate potential multi-locus interactions that predict each phenotype (after adjusting for significant covariates). Table 4 gives a summary of the significant results for models that had a testing accuracy over 0.591 (permutation *p*<0.05). Caffeine consumption, likelihood to doze, sleep duration on free days, minutes to get out of bed, off-shift sleep phase (corrected for sleep debt) and total work-week sleep duration were significantly predicted by SNP-SNP interactions. It is important to note that these multi-locus interactions were specific to shift type. The highest cross validation consistency (CVC) observed (4/5) were for multi-locus models that predicted sleep duration on free days (day-shifters only), minutes to get out of bed (night-shifters only), and off-shift sleep phase (corrected for sleep debt - day-shifters only). The MTNR1B.1 SNP in *MTNR1B* and the PER3.6 polymorphism in *PER3* interacted to predict sleep duration on days-off for those that work day-shift. Longer sleep duration on days off was associated with the combined effect of one allele of the rare variant in MTNR1B.1 and heterozygosity at the PER3.6 locus. In addition, AA-NAT.2 and PER3.6 jointly conferred off-shift sleep phase (corrected for sleep debt) for day-shifters such that carriers of the rare variant in AA-NAT.2 along with four repeats of PER3.6 on both alleles were more likely to have an earlier off-shift phase when on day shift. SNPs in four genes (AA-NAT.1, MTNR1B.2, CLOCK.2, and NPAS2.3) synergistically predicted the number of minutes necessary for night-shifters to get out of bed. Specifically, carriers with one or two alleles of the rare variant in NPAS2.3 but the common variant in each of the other three polymorphisms were associated with a reduction in the number of minutes to get out of bed (sleep inertia) when on night shift. Interestingly, almost all of these SNPs implicated in the multi-locus associations did not emerge in the single-locus analyses, which underscores the potential value of considering polygenic influences.

**Table 4:** Significant* multigenic models, testing accuracies, and cross-validation consistencies, identified by Generalized Multifactor Dimensionality Reduction (GMDR).

Shift	Outcome Variable	Model	Testing Accuracy	CVC
Day	Sleep Dur, Free Days	MTNR1B.1, PER3.6	0.60	4/5
Night	Min to get Out of Bed	AA-NAT.1, MTNR1B.2, CLOCK.2, NPAS2.3	0.65	4/5
Day	Quant. Chronotype	PER3.6, AA-NAT.2	0.62	4/5
Day	Sleep Dur, Free Days	AA-NAT.1, MTNR1B.3, PER3.6	0.61	3/5
Day	Min to get Out of Bed	CLOCK.2, AA-NAT.2	0.62	3/5
Day	Min to get Out of Bed	CLOCK.2, AA-NAT.2, NPAS2.1	0.62	3/5
Day	Caffeine	ARNTL2.5, CLOCK.2	0.62	2/5
Day	Caffeine	AA-NAT.1, CLOCK.2, PER3.6, ARNTL2.6	0.67	2/5
Day	Likelihood to Doze	AA-NAT.1, NPAS2.5	0.63	2/5
Day	Likelihood to Doze	PER3.6, AA-NAT.2, NPAS2.3, ARNTL2.6	0.63	2/5
Day	Min to get Out of Bed	AA-NAT.1, NPAS2.5, CLOCK.2, NPAS2.1	0.61	2/5
Day	Total Sleep Dur	MTNR1B.1, NPAS2.4, CLOCK.2	0.60	2/5
Day	Total Sleep Dur	CLOCK.2, PER3.6, AA-NAT.2, NPAS2.3	0.59	2/5
Day	Sleep Dur, Free Days	MTNR1B.1, NPAS2.4, CLOCK.2, PER3.6	0.62	1/5

*Multigenic models were considered significant if the testing testing accuracy was greater than 0.591 (permutation *p*<0.05).

## Discussion

According to the National Sleep Foundation 2008 poll, ∼7% of American workers are shift workers [40]. Previous research has associated shift work with an increased risk of developing cardiovascular disease, metabolic and gastrointestinal disorders, depression, and cancer [1], [2], [3], [4], it is not surprising that nurses in the present study do not adapt as well to a night-shift hospital schedule as to a day-shift. Shift work in hospital nurses is a particularly demanding example of shift-work disruption because (i) the shifts change from on to off so frequently and (ii) most night-shift nurses strive to flip immediately back to day-activity/night-sleep on days-off. This conclusion is especially valid for hospital nurses, since one out of four hospital nurses of all shift types report being excessively sleepy [41]. Night shift work within this population is associated with increased drug administration errors [41]. Furthermore, the long 12-h nurse shifts common in hospitals in the United States is potentially problematic, as suggested by an increased risk of patient mortality in hospitals with longer nurse work hours [42]. It is therefore not surprising that nurses in the present study did not adapt as well to a night-shift hospital schedule as to a day-shift. However, the underlying causes of this maladaptation that could become interventional targets are relatively unknown. Here, we present evidence that chronotype and newly defined sleep strategies contribute to adaptation to shift work. In addition, genotype is associated with caffeine consumption, chronotype, sleepiness, sleep inertia and sleep duration; however, some of these associations are dependent upon the “environment” of working shift.

Only a few studies have examined the influence of chronotype on the ability to cope with shift work schedules, and the results have been inconsistent. In rapidly rotating shift workers, morning (early) chronotypes were correlated with increased night-time drowsiness or chronic fatigue, while evening (late) chronotypes have been associated with better day-time sleep; however, day-shift versus night-shift comparisons were not made in these studies [43], [44]. Self-reported work performance is significantly predicted by whether a non-day-shift medical facility worker reports being either a “morning” or an “evening” type [45]. On the other hand, a more focused sample of nurses found that evening types were more likely to report sleep problems for day shift but there were no effects of chronotype for night shift [46]. In our sample of nurses, “adaptation” referred to levels of fatigue and regularity of sleep patterns (Figure S1). We found that earlier chronotypes (“larks”) had higher adaptation scores for day-shift and lower ones for night-shift, while later chronotypes (“owls”) had intermediate adaptation levels for both day and night shift. This result might be explained by a discrepancy between environmentally- imposed rest-activity cycles and the endogenous circadian clock [10], [47], [48], [49], [50], [51]. Therefore, the biggest discrepancies would be for larks working night-shift, with practically zero discrepancy for larks on day-shift. On the other hand, because owls have an intermediate biological time, there would be a smaller discrepancy for owls working night-shift or day-shift. Our results support this hypothesis as indicated by the regression lines for day-shift and night-shift converging at a late chronotype (∼4.5) rather than crossing at the middle chronotype of ∼3 (Figure 1D). Further, our finding that chronotype explains a smaller percentage of the variance in adaptation of night shift nurses compared to day shift nurses may explain why chronotype was unable to predict sleep problems in night shift workers in Newey et al [46].

A unique contribution of this study to the current shift work literature is the characterization of off-shift behavioral sleep strategies. Of the five strategies defined, we found that nurses who utilize sleep deprivation as a means to shift back to nocturnal sleep on days off (the “No Sleep” strategy) were the most poorly adapted, and that older and more experienced nurses were more likely to choose this strategy. Interestingly, ∼86% of larks chose to switch back to nocturnal sleep on days-off, and just over 1/3 of them chose the “No Sleep” strategy. The importance of nurses' work to general health is exemplified by the findings that over a quarter of hospital nurses suffer from excessive day-time sleepiness and that night-shift work is significantly associated with increased incidence of drug administration errors [41]. Our data provide evidence that arduous shift schedules which fragment the weekly sleep/wake patterns are detrimental to nurses' adaptation and possibly to their performance & alertness, which is likely to be necessary for good patient care.

Another putative contributing factor to shift work adjustment is the endogenous molecular clock mechanism. The 24-h timing of the molecular clock is orchestrated by an autoregulatory transcription/translation feedback loop composed of proteins encoded by the *CLOCK*, *NPAS2*, *BMAL1/2*, *PER*, and *CRY* genes [52], [53], [54], [55]. In our sample of nurses, a number of single-locus associations were observed. For example, two SNPs in *NPAS2* were associated with decreased likelihood to doze during the active period (Figure 4C) or later off-shift sleep phase (after correction for sleep debt) for day-shift nurses only (Figure 4F). Under night shift conditions, sleep phase may be sufficiently delayed for all genotypes such that the genotype-sleep phase association was not detected. In addition, polymorphisms in the hPER genes showed several associations in our study. For example, the well-studied coding-region variable number tandem repeat (VNTR) polymorphism in *PER3* (PER3.6) has been associated with morningness, delayed sleep phase syndrome, disrupted sleep architecture, and compromised cognitive performance [24], [56], [57], [58], [59], [60] but see also [61]. Those results suggested that genotype may confer an increased risk for medical professionals who routinely undergo sleep deprivation in order to meet work schedule demands [62], but our results suggest, in contrast, that nurses with five repeats of the PER3.6 polymorphism exhibit better adaptation when sleep deprivation was used as a strategy rather than long bouts of sleep (Figure 5D). Two additional SNPs in the *PER3* gene were linked in single-locus models to increased caffeine consumption in night-shift nurses only and decreased likelihood to doze in both shift types. Interestingly, one of these SNPs (PER3.7, aka rs10462021) was recently associated with greater daily sleep duration in a South Tyrolean population [63]. In addition to *PER3*, we found that a *PER2* SNP was associated with a day shift-specific lengthening of sleep duration over the 8-d work-cycle (Figure 4E). Validation of these results in independent samples and further functional studies are necessary to determine the reliability and precise role of these genetic variants on protein function and whether these changes ultimately affect circadian entrainment.

Human geneticists increasingly appreciate that most complex traits that have a genetic component are influenced not by just a single locus, but by complicated gene-gene interactions [64]. Our study is the first to analyze multi-locus effects for clock gene polymorphisms, and we found a number of significant gene-gene interactions (Table 4). For example, a SNP in the *CLOCK* gene (CLOCK.2, aka rs1801260) has been significantly associated with delayed sleep onset, reduced sleep, and insomnia [26], [65], and in our sample, this SNP was part of a multi-locus model that significantly predicted the number of minutes to get out of bed (sleep inertia) specifically for night shift nurses (Table 4). In addition, PER3.6 was part of two two-gene models that significantly conferred off-shift sleep phase (corrected for sleep debt) and off-shift sleep duration, respectively, in day-shift nurses (see Table 4).

This study of nurses on day- vs. night-shift provides a key example of how an environmental “stress” to the temporal organization of physiology and metabolism can have behavioral (and probably health-related [66], [67], [68], [69], [70]) consequences. In other systems, genotype (e.g., polymorphisms in the serotonin transporter gene, cannabinoid receptor 1, or catechol-O-methyltransferase) can significantly influence behavior such as depression, suicide attempts, hazardous drinking, and posttraumatic stress disorder, but only after an increased number of stressful life events or high traumatic load [71], [72], [73], [74]. These gene X environment interactions suggest that there is a genetic risk for developing anxiety and mood disorders, but only under stressful environmental conditions. The present results indicate a genetic risk for high caffeine consumption, excessive sleepiness, and longer weekly sleep duration, but only under shift-specific environments. For these night-shift specific associations, genetic influence on behavior may have been revealed by perturbation of the circadian system, as evidenced by the significant multi-locus model predicting the number of minutes to get out of bed. For day-shift specific associations, genetic influence on behavior may have been masked under night-shift conditions by the accompanying sleep disturbances and circadian misalignment. Thus, the present study has shown that circadian disruptions can act as environmental stressors to influence genetic risk. Specifically, chronotype, sleep strategy, and genotype interact to affect the ability of the circadian system to adjust to an environment that switches frequently and/or irregularly between different schedules based on the light-dark cycle and social/workplace time.

Our results also have health-policy implications. The ethical principles of nonmaleficence and beneficence prescribe that patients should be treated by health-care professionals who are not suffering from sleep deprivation. As in the case of residents working 30-consecutive hour shifts [62], sleep-deprived nurses might not be functioning at their highest capability [41]. At the very least, our studies of sleep strategy suggest that nurses should be informed that they should avoid sleep deprivation in anticipation of working the night shift (as in the “No Sleep” strategy). However, a more responsible approach would entail a re-evaluation of nurse schedule towards the possible reduction in the frequency of switching sleep schedules (as by staying more days on night-shift at a time followed by more days off) and/or reduction of the duration of night/evening shifts (e.g., 8 h instead of 12 h). Such a re-evaluation of 30-consecutive hour shifts for medical residents is underway [62].

## Supporting Information

Figure S1 **Sample survey administered to nurses.** The survey used in this study was based on The Munich Chronotype Questionnaire (MCQ; [27], which is used to assess chronotype by determining the mid-sleep time on days off. Because this method has not been used for shift workers, the MCQ was modified. Specifically, questions 14-19 and the self-assessment questionnaire were taken directly from the MCQ. In an effort to obtain more detailed information about sleep schedules on work days and free days, our survey included a typical schedule commonly used by Vanderbilt University Medical Center in which the nurses were instructed to shade in the 30-min time blocks in which they would normally be sleeping, including any naps. Some nurses had previous experience with similar shift schedules and could remember their sleep habits, and in those cases, the nurses filled out two schedules, one for nights and one for days, resulting in a total of 467 completed schedules. Of these, 88% either currently or previously worked a 7:00am/pm 12-h shift schedule.
(PDF)

Table S1 **Shift types represented in the sample.**
(DOC)

Table S2 **Genic location and minor allele frequencies (MAF) of candidate gene polymorphisms.**
(DOC)

Methods S1 (DOC)

## References

[1] Boivin DB, Tremblay GM, James FO (2007). Working on atypical schedules.. Sleep Med.

[2] Foster RG, Wulff K (2005). The rhythm of rest and excess.. Nat Rev Neurosci.

[3] Scheer FA, Hilton MF, Mantzoros CS, Shea SA (2009). Adverse metabolic and cardiovascular consequences of circadian misalignment.. Proc Natl Acad Sci U S A.

[4] Ardekani ZZ, Kakooei H, Ayattollahi SM, Choobineh A, Seraji GN (2008). Prevalence of mental disorders among shift work hospital nurses in Shiraz, Iran.. Pak J Biol Sci.

[5] Chen JD, Lin YC, Hsiao ST (2010). Obesity and high blood pressure of 12-hour night shift female clean-room workers.. Chronobiol Int.

[6] Marino JL, Holt VL, Chen C, Davis S (2008). Shift work, hCLOCK T3111C polymorphism, and endometriosis risk.. Epidemiology.

[7] Schernhammer ES, Laden F, Speizer FE, Willett WC, Hunter DJ (2001). Rotating night shifts and risk of breast cancer in women participating in the nurses' health study.. J Natl Cancer Inst.

[8] Lin YC, Hsiao TJ, Chen PC (2009). Shift work aggravates metabolic syndrome development among early-middle-aged males with elevated ALT.. World J Gastroenterol.

[9] Pietroiusti A, Neri A, Somma G, Coppeta L, Iavicoli I (2010). Incidence of metabolic syndrome among night-shift healthcare workers.. Occup Environ Med.

[10] Wittmann M, Dinich J, Merrow M, Roenneberg T (2006). Social jetlag: misalignment of biological and social time.. Chronobiol Int.

[11] Lee ML, Swanson BE, de la Iglesia HO (2009). Circadian timing of REM sleep is coupled to an oscillator within the dorsomedial suprachiasmatic nucleus.. Curr Biol.

[12] Yamazaki S, Numano R, Abe M, Hida A, Takahashi R (2000). Resetting central and peripheral circadian oscillators in transgenic rats.. Science.

[13] Davidson AJ, Sellix MT, Daniel J, Yamazaki S, Menaker M (2006). Chronic jet-lag increases mortality in aged mice.. Curr Biol.

[14] Castanon-Cervantes O, Wu M, Ehlen JC, Paul KN, Gamble KL (2010). Disregulation of inflammatory responses by chronic circadian disruption..

[15] Yan J, Wang H, Liu Y, Shao C (2008). Analysis of gene regulatory networks in the mammalian circadian rhythm.. PLoS Comput Biol.

[16] Mendlewicz J (2009). Disruption of the circadian timing systems: molecular mechanisms in mood disorders.. CNS Drugs.

[17] Artioli P, Lorenzi C, Pirovano A, Serretti A, Benedetti F (2007). How do genes exert their role? Period 3 gene variants and possible influences on mood disorder phenotypes.. Eur Neuropsychopharmacol.

[18] Partonen T, Treutlein J, Alpman A, Frank J, Johansson C (2007). Three circadian clock genes Per2, Arntl, and Npas2 contribute to winter depression.. Ann Med.

[19] Takao T, Tachikawa H, Kawanishi Y, Mizukami K, Asada T (2007). CLOCK gene T3111C polymorphism is associated with Japanese schizophrenics: a preliminary study.. Eur Neuropsychopharmacol.

[20] Nicholas B, Rudrasingham V, Nash S, Kirov G, Owen MJ (2007). Association of Per1 and Npas2 with autistic disorder: support for the clock genes/social timing hypothesis.. Mol Psychiatry.

[21] Englund A, Kovanen L, Saarikoski ST, Haukka J, Reunanen A (2009). NPAS2 and PER2 are linked to risk factors of the metabolic syndrome.. J Circadian Rhythms.

[22] Wulff K, Porcheret K, Cussans E, Foster RG (2009). Sleep and circadian rhythm disturbances: multiple genes and multiple phenotypes.. Curr Opin Genet Dev.

[23] Carpen JD, von Schantz M, Smits M, Skene DJ, Archer SN (2006). A silent polymorphism in the PER1 gene associates with extreme diurnal preference in humans.. J Hum Genet.

[24] Archer SN, Robilliard DL, Skene DJ, Smits M, Williams A (2003). A length polymorphism in the circadian clock gene Per3 is linked to delayed sleep phase syndrome and extreme diurnal preference.. Sleep.

[25] Toh KL, Jones CR, He Y, Eide EJ, Hinz WA (2001). An hPer2 phosphorylation site mutation in familial advanced sleep phase syndrome.. Science.

[26] Benedetti F, Dallaspezia S, Fulgosi MC, Lorenzi C, Serretti A (2007). Actimetric evidence that CLOCK 3111 T/C SNP influences sleep and activity patterns in patients affected by bipolar depression.. Am J Med Genet B Neuropsychiatr Genet.

[27] Roenneberg T, Wirz-Justice A, Merrow M (2003). Life between clocks: daily temporal patterns of human chronotypes.. J Biol Rhythms.

[28] Roenneberg T, Kuehnle T, Pramstaller PP, Ricken J, Havel M (2004). A marker for the end of adolescence.. Curr Biol.

[29] Ciarleglio CM, Ryckman KK, Servick SV, Hida A, Robbins S (2008). Genetic differences in human circadian clock genes among worldwide populations.. J Biol Rhythms.

[30] Nataraj AJ, Olivos-Glander I, Kusukawa N, Highsmith WE (1999). Single-strand conformation polymorphism and heteroduplex analysis for gel-based mutation detection.. Electrophoresis.

[31] Lou XY, Chen GB, Yan L, Ma JZ, Zhu J (2007). A generalized combinatorial approach for detecting gene-by-gene and gene-by-environment interactions with application to nicotine dependence.. Am J Hum Genet.

[32] Rogers AE, Caruso CC, Aldrich MS (1993). Reliability of sleep diaries for assessment of sleep/wake patterns.. Nurs Res.

[33] Monk TH, Buysse DJ, Kennedy KS, Pods JM, DeGrazia JM (2003). Measuring sleep habits without using a diary: the sleep timing questionnaire.. Sleep.

[34] Crowley SJ, Lee C, Tseng CY, Fogg LF, Eastman CI (2004). Complete or partial circadian re-entrainment improves performance, alertness, and mood during night-shift work.. Sleep.

[35] Lee C, Smith MR, Eastman CI (2006). A compromise phase position for permanent night shift workers: circadian phase after two night shifts with scheduled sleep and light/dark exposure.. Chronobiol Int.

[36] Smith MR, Eastman CI (2008). Night shift performance is improved by a compromise circadian phase position: study 3. Circadian phase after 7 night shifts with an intervening weekend off.. Sleep.

[37] Smith MR, Fogg LF, Eastman CI (2009). Practical interventions to promote circadian adaptation to permanent night shift work: study 4.. J Biol Rhythms.

[38] von Schantz M (2008). Phenotypic effects of genetic variability in human clock genes on circadian and sleep parameters.. J Genet.

[39] Gibson G (2008). The environmental contribution to gene expression profiles.. Nat Rev Genet.

[40] (2008). 2008 Sleep in America Poll Washington, DC: National Sleep Foundation.

[41] Suzuki K, Ohida T, Kaneita Y, Yokoyama E, Uchiyama M (2005). Daytime sleepiness, sleep habits and occupational accidents among hospital nurses.. J Adv Nurs.

[42] Trinkoff AM, Johantgen M, Storr CL, Gurses AP, Liang Y (2011). Nurses' work schedule characteristics, nurse staffing, and patient mortality.. Nurs Res.

[43] Taylor E, Folkard S, Shapiro DA (1997). Shiftwork Advantages as Predictors of Health.. Int J Occup Environ Health.

[44] Smith L, Tanigawa T, Takahashi M, Mutou K, Tachibana N (2005). Shiftwork locus of control, situational and behavioural effects on sleepiness and fatigue in shiftworkers.. Ind Health.

[45] Burch JB, Tom J, Zhai Y, Criswell L, Leo E (2009). Shiftwork impacts and adaptation among health care workers.. Occup Med (Lond).

[46] Newey CA, Hood BM (2004). Determinants of shift-work adjustment for nursing staff: the critical experience of partners.. J Prof Nurs.

[47] Lund J, Arendt J, Hampton SM, English J, Morgan LM (2001). Postprandial hormone and metabolic responses amongst shift workers in Antarctica.. J Endocrinol.

[48] Weibel L, Spiegel K, Gronfier C, Follenius M, Brandenberger G (1997). Twenty-four-hour melatonin and core body temperature rhythms: their adaptation in night workers.. Am J Physiol.

[49] Boivin DB, James FO (2002). Circadian adaptation to night-shift work by judicious light and darkness exposure.. J Biol Rhythms.

[50] Hennig J, Kieferdorf P, Moritz C, Huwe S, Netter P (1998). Changes in cortisol secretion during shiftwork: implications for tolerance to shiftwork?. Ergonomics.

[51] James FO, Boivin DB, Charbonneau S, Bélanger V, Cermakian N (2007). Expression of Clock Genes in Human Peripheral Blood Mononuclear Cells throughout the Sleep/Wake and Circadian Cycles.. Chronobiology International: The Journal of Biological & Medical Rhythm Research.

[52] Takahashi JS, Hong HK, Ko CH, McDearmon EL (2008). The genetics of mammalian circadian order and disorder: implications for physiology and disease.. Nat Rev Genet.

[53] Debruyne JP, Noton E, Lambert CM, Maywood ES, Weaver DR (2006). A clock shock: mouse CLOCK is not required for circadian oscillator function.. Neuron.

[54] Dudley CA, Erbel-Sieler C, Estill SJ, Reick M, Franken P (2003). Altered patterns of sleep and behavioral adaptability in NPAS2-deficient mice.. Science.

[55] Pendergast JS, Friday RC, Yamazaki S (2010). Distinct functions of Period2 and Period3 in the mouse circadian system revealed by in vitro analysis.. PLoS One.

[56] Jones KH, Ellis J, von Schantz M, Skene DJ, Dijk DJ (2007). Age-related change in the association between a polymorphism in the PER3 gene and preferred timing of sleep and waking activities.. J Sleep Res.

[57] Ebisawa T, Uchiyama M, Kajimura N, Mishima K, Kamei Y (2001). Association of structural polymorphisms in the human period3 gene with delayed sleep phase syndrome.. EMBO Rep.

[58] Pereira DS, Tufik S, Louzada FM, Benedito-Silva AA, Lopez AR (2005). Association of the length polymorphism in the human Per3 gene with the delayed sleep-phase syndrome: does latitude have an influence upon it?. Sleep.

[59] Groeger JA, Viola AU, Lo JC, von Schantz M, Archer SN (2008). Early morning executive functioning during sleep deprivation is compromised by a PERIOD3 polymorphism.. Sleep.

[60] Viola AU, Archer SN, James LM, Groeger JA, Lo JC (2007). PER3 polymorphism predicts sleep structure and waking performance.. Curr Biol.

[61] Goel N, Banks S, Mignot E, Dinges DF (2009). PER3 polymorphism predicts cumulative sleep homeostatic but not neurobehavioral changes to chronic partial sleep deprivation.. PLoS One.

[62] Czeisler CA (2009). Medical and genetic differences in the adverse impact of sleep loss on performance: ethical considerations for the medical profession.. Trans Am Clin Climatol Assoc.

[63] Allebrandt KV, Teder-Laving M, Akyol M, Pichler I, Muller-Myhsok B (2010). CLOCK Gene Variants Associate with Sleep Duration in Two Independent Populations..

[64] Moore JH (2003). The ubiquitous nature of epistasis in determining susceptibility to common human diseases.. Hum Hered.

[65] Serretti A, Benedetti F, Mandelli L, Lorenzi C, Pirovano A (2003). Genetic dissection of psychopathological symptoms: insomnia in mood disorders and CLOCK gene polymorphism.. Am J Med Genet B Neuropsychiatr Genet.

[66] Karlsson B, Knutsson A, Lindahl B (2001). Is there an association between shift work and having a metabolic syndrome? Results from a population based study of 27,485 people.. Occup Environ Med.

[67] Schernhammer ES, Laden F, Speizer FE, Willett WC, Hunter DJ (2003). Night-shift work and risk of colorectal cancer in the nurses' health study.. J Natl Cancer Inst.

[68] Knutsson A (2003). Health disorders of shift workers.. Occup Med (Lond).

[69] Janssen D, Nachreiner F (2004). Health and psychosocial effects of flexible working hours.. Rev Saude Publica.

[70] Kaliterna LL, Prizmic LZ, Zganec N (2004). Quality of life, life satisfaction and happiness in shift- and non-shiftworkers.. Rev Saude Publica.

[71] Caspi A, Sugden K, Moffitt TE, Taylor A, Craig IW (2003). Influence of life stress on depression: moderation by a polymorphism in the 5-HTT gene.. Science.

[72] Laucht M, Treutlein J, Schmid B, Blomeyer D, Becker K (2009). Impact of psychosocial adversity on alcohol intake in young adults: moderation by the LL genotype of the serotonin transporter polymorphism.. Biol Psychiatry.

[73] Juhasz G, Chase D, Pegg E, Downey D, Toth ZG (2009). CNR1 gene is associated with high neuroticism and low agreeableness and interacts with recent negative life events to predict current depressive symptoms.. Neuropsychopharmacology.

[74] Kolassa IT, Kolassa S, Ertl V, Papassotiropoulos A, De Quervain DJ (2010). The risk of posttraumatic stress disorder after trauma depends on traumatic load and the catechol-o-methyltransferase Val(158)Met polymorphism.. Biol Psychiatry.

