# Supplementary material for: Shift Work in Nurses: Contribution of Phenotypes and Genotypes to Adaptation
Source: PLoS One. 2011 Apr 13;6(4):e18395. doi: 10.1371/journal.pone.0018395 (PMC3076422; doi:10.1371/journal.pone.0018395)
Supplement: Figure S1 — Sample survey administered to nurses. The survey used in this study was based on The Munich Chronotype Questionnaire (MCQ; [27], which is used to assess chronotype by determining the mid-sleep time on days off. Because this method has not been used for shift workers, the MCQ was modified. Specifically, questions 14-19 and the self-assessment questionnaire were taken directly from the MCQ. In an effort to obtain more detailed information about sleep schedules on work days and free days, our survey included a typical schedule commonly used by Vanderbilt University Medical Center in which the nurses were instructed to shade in the 30-min time blocks in which they would normally be sleeping, including any naps. Some nurses had previous experience with similar shift schedules and could remember their sleep habits, and in those cases, the nurses filled out two schedules, one for nights and one for days, resulting in a total of 467 completed schedules. Of these, 88% either currently or previously worked a 7:00am/pm 12-h shift schedule. (PDF) [file pone.0018395.s001.pdf]

Biological Clocks Questionnaire

People arrange their daily lives differently according to when they go to bed and wake up (also known as an individual's "clock gene" network). In our research, we are trying to find the biological mechanisms behind the phenomenon of different clock genes. What makes a person a night owl? Why are some people early risers? We are particularly interested in how people with different clock gene networks adapt to shift work.

On this page, please fill out you personal and contact information. This information will be kept confidential at all times. We ask for your name, address and contact information in case we wish to contact you for participation in future research. Please be assured that all of your personal information will be treated with the utmost confidentiality.

After you have completed this questionnaire, it will be encoded with a unique number, and the front page—with all of your identifying information—will be removed and stored separately in a locked cabinet in the office of Nursing Research. All evaluations will be performed only with the anonymous, encoded pages that follow, and **at no time** will any of your identifying information be associated with the answers you give throughout the questionnaire.

Name: ____

Address: ____

____

____

Telephone: ____

E-Mail: ____

CODE NUMBER (please do not fill out): ________________________________

NOTE: On each subsequent page, you will see a space for the above code number, generated by research personnel. Please leave each of these spaces blank).

This questionnaire is based on a questionnaire originally designed by Drs. Till Roenneberg and Martha Merrow in Munich, Germany and Dr. Anna Wirz-Justice in Basel, Switzerland.

CODE NUMBER __________

1. Which hours do you currently work?
___7am-7pm ___7pm-7am ___7am-3pm ___3pm-11pm ___11pm-7am
___Other "daytime" 12-hour shift (example: 4am-4pm)
___Other "nighttime" 12-hour shift (example: 4pm-4am)
___Other daytime 8-hour shift (example: 4am-12:00pm)
___Other nighttime 8-hour shift (example: 12:00 pm-8:00pm)
___Other hospital shift hours (please give shift times):______________________
___I do not work in hospital shift hours; I work "normal" clinic/office hours

2. How many months/years have you worked these current hours?
___0-11 months ___1-2 years ___3-5 years ___6-10 years ___more than 10 years

3. Do you routinely alternate the hours you work between daytime and nighttime hours (at least once every two weeks?) ___yes ___no

4. On a scale from 1-10, how well do you feel you adapt to your current work hours? (Answer by circling the appropriate number on the grid below):
Examples: 1 = not well at all; I tend to feel tired all the time, cannot enjoy my days off, and my sleep cycles never seem to be regulated
5 = middle-of-the-road; I'm okay with working this shift, but I still feel tired on my first day off, and my sleep patterns vary at times
10 = very well; I really enjoy this shift, have no trouble getting my energy back on my first days off, and sleep just as well when working as I do when not working

1	2	3	4	5	6	7	8	9	10
not well				middle-of-the-road					very well

If you are not currently a hospital shift worker (i.e., day or night shift), but you have previous experience as a hospital shift worker in another position, please answer questions 5-9. If you are currently a hospital shift worker, or you have never worked hospital shifts, please proceed to question 10.

5. Which hours did you work? (choose only one option; if you've worked in more than one position as a shift worker, or more than one type of shift, please refer to your most recent position):
___7am-7pm ___7pm-7am ___7am-3pm ___3pm-11pm ___11pm-7am
___Other daytime 12-hour shift (example: 4am-4pm)
___Other nighttime 12-hour shift (example: 4pm-4am)
___Other daytime 8-hour shift (example: 4am-12:00pm)
___Other nighttime 8-hour shift (example: 12:00 pm-8:00pm)
___Other hospital shift hours (please give shift times): ______________________

6. How many months/years did you work these shift hours?
___0-11 months ___1-2 years ___3-5 years ___6-10 years ___more than 10 years

7. Did you routinely alternate the hours you worked between daytime and nighttime hours (at least once every two weeks?) ___yes ___no

8. On a scale from 1-10, how well do you feel you adapted to these shift hours? (answer by circling the appropriate number on the grid below):

Examples: 1 = not well at all; I tended to feel tired all the time, cannot enjoy my days off, and my sleep cycles never seemed to be regulated

5 = middle-of-the-road; I was okay with working this shift, but I still felt tired on my first day off, and my sleep patterns varied at times

10 = very well; I really enjoyed this shift, had no trouble getting my energy back on my first days off, and slept just as well when working as I did when not working

1	2	3	4	5	6	7	8	9	10
not well				middle-of-the-road					very well

9. Why did you leave shift work for your current position? (check all that apply);
___could not adapt to shift work
___other (please specify): ____

10. How likely are you to doze off (rather than just feeling tired) while sitting and reading or watching TV? Rate as: “never doze” (ND); “slight chance” (S); “moderate chance” (M); or “high chance” (H) of dozing.

____

11. How many caffeinated drinks do you drink daily? (Please answer in terms of the number of cups of coffee or tea or number of portions of caffeinated soft drinks per day):

____

12. What is your average alcohol intake? (Please answer in terms of the number of alcohol-containing drinks per week):

____

13. Are you regularly taking any medications that might affect your sleep patterns?
___ yes ___no

The charts on the next two pages represent a *hypothetical* work-week with five days off and three days on for a night shift or a day shift schedule. In this sample week, days off are typical days off without parties or travel. Working times are shown in grey. If you are a night shift worker, please fill in the night shift chart (page 4) to document your typical sleeping patterns. If you are a day shift worker, please fill in the day shift chart (page 5) to document your typical sleeping patterns. If you have worked both shifts and you remember both of your patterns accurately, please fill in both charts–if you can't remember both patterns, fill in the one you remember best (probably your current shift).

Table 1: Night Shift

Day A	Day B	Day C	Day D	Day E	Day F	Day G	Day H
Off	Off	Off	Off Wo	rk Wo	rk Wo	rk Off	Off

Please clearly and **precisely** indicate the times you sleep and/or regularly nap during this hypothetical week by blacking out the relevant squares.

Day A	Day B	Day C	Day D	Day E	Day F	Day G	Day H
12-12:30 a.m.	12-12:30 a.m.	12-12:30 a.m.	12-12:30 a.m.	12-12:30 a.m.	12-12:30 a.m.	12-12:30 a.m.	12-12:30 a.m.
12:30-1 a.m.	12:30-1 a.m.	12:30-1 a.m.	12:30-1 a.m.	12:30-1 a.m.	12:30-1 a.m.	12:30-1 a.m.	12:30-1 a.m.
1-1:30 a.m.	1-1:30 a.m.	1-1:30 a.m.	1-1:30 a.m.	1-1:30 a.m.	1-1:30 a.m.	1-1:30 a.m.	1-1:30 a.m.
1:30-2 a.m.	1:30-2 a.m.	1:30-2 a.m.	1:30-2 a.m.	1:30-2 a.m.	1:30-2 a.m.	1:30-2 a.m.	1:30-2 a.m.
2-2:30 a.m.	2-2:30 a.m.	2-2:30 a.m.	2-2:30 a.m.	2-2:30 a.m.	2-2:30 a.m.	2-2:30 a.m.	2-2:30 a.m.
2:30-3 a.m.	2:30-3 a.m.	2:30-3 a.m.	2:30-3 a.m.	2:30-3 a.m.	2:30-3 a.m.	2:30-3 a.m.	2:30-3 a.m.
3-3:30 a.m.	3-3:30 a.m.	3-3:30 a.m.	3-3:30 a.m.	3-3:30 a.m.	3-3:30 a.m.	3-3:30 a.m.	3-3:30 a.m.
3:30-4 a.m.	3:30-4 a.m.	3:30-4 a.m.	3:30-4 a.m.	3:30-4 a.m.	3:30-4 a.m.	3:30-4 a.m.	3:30-4 a.m.
4-4:30 a.m.	4-4:30 a.m.	4-4:30 a.m.	4-4:30 a.m.	4-4:30 a.m.	4-4:30 a.m.	4-4:30 a.m.	4-4:30 a.m.
4:30-5 a.m.	4:30-5 a.m.	4:30-5 a.m.	4:30-5 a.m.	4:30-5 a.m.	4:30-5 a.m.	4:30-5 a.m.	4:30-5 a.m.
5-5:30 a.m.	5-5:30 a.m.	5-5:30 a.m.	5-5:30 a.m.	5-5:30 a.m.	5-5:30 a.m.	5-5:30 a.m.	5-5:30 a.m.
5:30-6 a.m.	5:30-6 a.m.	5:30-6 a.m.	5:30-6 a.m.	5:30-6 a.m.	5:30-6 a.m.	5:30-6 a.m.	5:30-6 a.m.
6-6:30 a.m.	6-6:30 a.m.	6-6:30 a.m.	6-6:30 a.m.	6-6:30 a.m.	6-6:30 a.m.	6-6:30 a.m.	6-6:30 a.m.
6:30-7 a.m.	6:30-7 a.m.	6:30-7 a.m.	6:30-7 a.m.	6:30-7 a.m.	6:30-7 a.m.	6:30-7 a.m.	6:30-7 a.m.
7-7:30 a.m.	7-7:30 a.m.	7-7:30 a.m.	7-7:30 a.m.	7-7:30 a.m.	7-7:30 a.m.	7-7:30 a.m.	7-7:30 a.m.
7:30-8 a.m.	7:30-8 a.m.	7:30-8 a.m.	7:30-8 a.m.	7:30-8 a.m.	7:30-8 a.m.	7:30-8 a.m.	7:30-8 a.m.
8-8:30 a.m.	8-8:30 a.m.	8-8:30 a.m.	8-8:30 a.m.	8-8:30 a.m.	8-8:30 a.m.	8-8:30 a.m.	8-8:30 a.m.
8:30-9 a.m.	8:30-9 a.m.	8:30-9 a.m.	8:30-9 a.m.	8:30-9 a.m.	8:30-9 a.m.	8:30-9 a.m.	8:30-9 a.m.
9-9:30 a.m.	9-9:30 a.m.	9-9:30 a.m.	9-9:30 a.m.	9-9:30 a.m.	9-9:30 a.m.	9-9:30 a.m.	9-9:30 a.m.
9:30-10 a.m.	9:30-10 a.m.	9:30-10 a.m.	9:30-10 a.m.	9:30-10 a.m.	9:30-10 a.m.	9:30-10 a.m.	9:30-10 a.m.
10-10:30 a.m.	10-10:30 a.m.	10-10:30 a.m.	10-10:30 a.m.	10-10:30 a.m.	10-10:30 a.m.	10-10:30 a.m.	10-10:30 a.m.
10:30-11 a.m.	10:30-11 a.m.	10:30-11 a.m.	10:30-11 a.m.	10:30-11 a.m.	10:30-11 a.m.	10:30-11 a.m.	10:30-11 a.m.
11-11:30 a.m.	11-11:30 a.m.	11-11:30 a.m.	11-11:30 a.m.	11-11:30 a.m.	11-11:30 a.m.	11-11:30 a.m.	11-11:30 a.m.
11:30-12 p.m.	11:30-12 p.m.	11:30-12 p.m.	11:30-12 p.m.	11:30-12 p.m.	11:30-12 p.m.	11:30-12 p.m.	11:30-12 p.m.
12-12:30 p.m.	12-12:30 p.m.	12-12:30 p.m.	12-12:30 p.m.	12-12:30 p.m.	12-12:30 p.m.	12-12:30 p.m.	12-12:30 p.m.
12:30-1 p.m.	12:30-1 p.m.	12:30-1 p.m.	12:30-1 p.m.	12:30-1 p.m.	12:30-1 p.m.	12:30-1 p.m.	12:30-1 p.m.
1-1:30 p.m.	1-1:30 p.m.	1-1:30 p.m.	1-1:30 p.m.	1-1:30 p.m.	1-1:30 p.m.	1-1:30 p.m.	1-1:30 p.m.
1:30-2 p.m.	1:30-2 p.m.	1:30-2 p.m.	1:30-2 p.m.	1:30-2 p.m.	1:30-2 p.m.	1:30-2 p.m.	1:30-2 p.m.
2-2:30 p.m.	2-2:30 p.m.	2-2:30 p.m.	2-2:30 p.m.	2-2:30 p.m.	2-2:30 p.m.	2-2:30 p.m.	2-2:30 p.m.
2:30-3 p.m.	2:30-3 p.m.	2:30-3 p.m.	2:30-3 p.m.	2:30-3 p.m.	2:30-3 p.m.	2:30-3 p.m.	2:30-3 p.m.
3-3:30 p.m.	3-3:30 p.m.	3-3:30 p.m.	3-3:30 p.m.	3-3:30 p.m.	3-3:30 p.m.	3-3:30 p.m.	3-3:30 p.m.
3:30-4 p.m.	3:30-4 p.m.	3:30-4 p.m.	3:30-4 p.m.	3:30-4 p.m.	3:30-4 p.m.	3:30-4 p.m.	3:30-4 p.m.
4-4:30 p.m.	4-4:30 p.m.	4-4:30 p.m.	4-4:30 p.m.	4-4:30 p.m.	4-4:30 p.m.	4-4:30 p.m.	4-4:30 p.m.
4:30-5 p.m.	4:30-5 p.m.	4:30-5 p.m.	4:30-5 p.m.	4:30-5 p.m.	4:30-5 p.m.	4:30-5 p.m.	4:30-5 p.m.
5-5:30 p.m.	5-5:30 p.m.	5-5:30 p.m.	5-5:30 p.m.	5-5:30 p.m.	5-5:30 p.m.	5-5:30 p.m.	5-5:30 p.m.
5:30-6 p.m.	5:30-6 p.m.	5:30-6 p.m.	5:30-6 p.m.	5:30-6 p.m.	5:30-6 p.m.	5:30-6 p.m.	5:30-6 p.m.
6-6:30 p.m.	6-6:30 p.m.	6-6:30 p.m.	6-6:30 p.m.	6-6:30 p.m.	6-6:30 p.m.	6-6:30 p.m.	6-6:30 p.m.
6:30-7 p.m.	6:30-7 p.m.	6:30-7 p.m.	6:30-7 p.m.	6:30-7 p.m.	6:30-7 p.m.	6:30-7 p.m.	6:30-7 p.m.
7-7:30 p.m.	7-7:30 p.m.	7-7:30 p.m.	7-7:30 p.m.	7-7:30 p.m.	7-7:30 p.m.	7-7:30 p.m.	7-7:30 p.m.
7:30-8 p.m.	7:30-8 p.m.	7:30-8 p.m.	7:30-8 p.m.	7:30-8 p.m.	7:30-8 p.m.	7:30-8 p.m.	7:30-8 p.m.
8-8:30 p.m.	8-8:30 p.m.	8-8:30 p.m.	8-8:30 p.m.	8-8:30 p.m.	8-8:30 p.m.	8-8:30 p.m.	8-8:30 p.m.
8:30-9 p.m.	8:30-9 p.m.	8:30-9 p.m.	8:30-9 p.m.	8:30-9 p.m.	8:30-9 p.m.	8:30-9 p.m.	8:30-9 p.m.
9-9:30 p.m.	9-9:30 p.m.	9-9:30 p.m.	9-9:30 p.m.	9-9:30 p.m.	9-9:30 p.m.	9-9:30 p.m.	9-9:30 p.m.
9:30-10 p.m.	9:30-10 p.m.	9:30-10 p.m.	9:30-10 p.m.	9:30-10 p.m.	9:30-10 p.m.	9:30-10 p.m.	9:30-10 p.m.
10-10:30 p.m.	10-10:30 p.m.	10-10:30 p.m.	10-10:30 p.m.	10-10:30 p.m.	10-10:30 p.m.	10-10:30 p.m.	10-10:30 p.m.
10:30-11 p.m.	10:30-11 p.m.	10:30-11 p.m.	10:30-11 p.m.	10:30-11 p.m.	10:30-11 p.m.	10:30-11 p.m.	10:30-11 p.m.
11-11:30 p.m.	11-11:30 p.m.	11-11:30 p.m.	11-11:30 p.m.	11-11:30 p.m.	11-11:30 p.m.	11-11:30 p.m.	11-11:30 p.m.
11:30-12 a.m.	11:30-12 a.m.	11:30-12 a.m.	11:30-12 a.m.	11:30-12 a.m.	11:30-12 a.m.	11:30-12 a.m.	11:30-12 a.m.

CODE NUMBER: __________

Table 2: Day Shift

Day A	Day B	Day C	Day D	Day E	Day F	Day G	Day H
Off	Off	Off	Work	Work	Work	Off	Off

Please clearly and **precisely** indicate the times you sleep and/or regularly nap during this hypothetical week by blacking out the relevant squares.

Day A	Day B	Day C	Day D	Day E	Day F	Day G	Day H
12-12:30 a.m.	12-12:30 a.m.	12-12:30 a.m.	12-12:30 a.m.	12-12:30 a.m.	12-12:30 a.m.	12-12:30 a.m.	12-12:30 a.m.
12:30-1 a.m.	12:30-1 a.m.	12:30-1 a.m.	12:30-1 a.m.	12:30-1 a.m.	12:30-1 a.m.	12:30-1 a.m.	12:30-1 a.m.
1-1:30 a.m.	1-1:30 a.m.	1-1:30 a.m.	1-1:30 a.m.	1-1:30 a.m.	1-1:30 a.m.	1-1:30 a.m.	1-1:30 a.m.
1:30-2 a.m.	1:30-2 a.m.	1:30-2 a.m.	1:30-2 a.m.	1:30-2 a.m.	1:30-2 a.m.	1:30-2 a.m.	1:30-2 a.m.
2-2:30 a.m.	2-2:30 a.m.	2-2:30 a.m.	2-2:30 a.m.	2-2:30 a.m.	2-2:30 a.m.	2-2:30 a.m.	2-2:30 a.m.
2:30-3 a.m.	2:30-3 a.m.	2:30-3 a.m.	2:30-3 a.m.	2:30-3 a.m.	2:30-3 a.m.	2:30-3 a.m.	2:30-3 a.m.
3-3:30 a.m.	3-3:30 a.m.	3-3:30 a.m.	3-3:30 a.m.	3-3:30 a.m.	3-3:30 a.m.	3-3:30 a.m.	3-3:30 a.m.
3:30-4 a.m.	3:30-4 a.m.	3:30-4 a.m.	3:30-4 a.m.	3:30-4 a.m.	3:30-4 a.m.	3:30-4 a.m.	3:30-4 a.m.
4-4:30 a.m.	4-4:30 a.m.	4-4:30 a.m.	4-4:30 a.m.	4-4:30 a.m.	4-4:30 a.m.	4-4:30 a.m.	4-4:30 a.m.
4:30-5 a.m.	4:30-5 a.m.	4:30-5 a.m.	4:30-5 a.m.	4:30-5 a.m.	4:30-5 a.m.	4:30-5 a.m.	4:30-5 a.m.
5-5:30 a.m.	5-5:30 a.m.	5-5:30 a.m.	5-5:30 a.m.	5-5:30 a.m.	5-5:30 a.m.	5-5:30 a.m.	5-5:30 a.m.
5:30-6 a.m.	5:30-6 a.m.	5:30-6 a.m.	5:30-6 a.m.	5:30-6 a.m.	5:30-6 a.m.	5:30-6 a.m.	5:30-6 a.m.
6-6:30 a.m.	6-6:30 a.m.	6-6:30 a.m.	6-6:30 a.m.	6-6:30 a.m.	6-6:30 a.m.	6-6:30 a.m.	6-6:30 a.m.
6:30-7 a.m.	6:30-7 a.m.	6:30-7 a.m.	6:30-7 a.m.	6:30-7 a.m.	6:30-7 a.m.	6:30-7 a.m.	6:30-7 a.m.
7-7:30 a.m.	7-7:30 a.m.	7-7:30 a.m.	7-7:30 a.m.	7-7:30 a.m.	7-7:30 a.m.	7-7:30 a.m.	7-7:30 a.m.
7:30-8 a.m.	7:30-8 a.m.	7:30-8 a.m.	7:30-8 a.m.	7:30-8 a.m.	7:30-8 a.m.	7:30-8 a.m.	7:30-8 a.m.
8-8:30 a.m.	8-8:30 a.m.	8-8:30 a.m.	8-8:30 a.m.	8-8:30 a.m.	8-8:30 a.m.	8-8:30 a.m.	8-8:30 a.m.
8:30-9 a.m.	8:30-9 a.m.	8:30-9 a.m.	8:30-9 a.m.	8:30-9 a.m.	8:30-9 a.m.	8:30-9 a.m.	8:30-9 a.m.
9-9:30 a.m.	9-9:30 a.m.	9-9:30 a.m.	9-9:30 a.m.	9-9:30 a.m.	9-9:30 a.m.	9-9:30 a.m.	9-9:30 a.m.
9:30-10 a.m.	9:30-10 a.m.	9:30-10 a.m.	9:30-10 a.m.	9:30-10 a.m.	9:30-10 a.m.	9:30-10 a.m.	9:30-10 a.m.
10-10:30 a.m.	10-10:30 a.m.	10-10:30 a.m.	10-10:30 a.m.	10-10:30 a.m.	10-10:30 a.m.	10-10:30 a.m.	10-10:30 a.m.
10:30-11 a.m.	10:30-11 a.m.	10:30-11 a.m.	10:30-11 a.m.	10:30-11 a.m.	10:30-11 a.m.	10:30-11 a.m.	10:30-11 a.m.
11-11:30 a.m.	11-11:30 a.m.	11-11:30 a.m.	11-11:30 a.m.	11-11:30 a.m.	11-11:30 a.m.	11-11:30 a.m.	11-11:30 a.m.
11:30-12 p.m.	11:30-12 p.m.	11:30-12 p.m.	11:30-12 p.m.	11:30-12 p.m.	11:30-12 p.m.	11:30-12 p.m.	11:30-12 p.m.
12-12:30 p.m.	12-12:30 p.m.	12-12:30 p.m.	12-12:30 p.m.	12-12:30 p.m.	12-12:30 p.m.	12-12:30 p.m.	12-12:30 p.m.
12:30-1 p.m.	12:30-1 p.m.	12:30-1 p.m.	12:30-1 p.m.	12:30-1 p.m.	12:30-1 p.m.	12:30-1 p.m.	12:30-1 p.m.
1-1:30 p.m.	1-1:30 p.m.	1-1:30 p.m.	1-1:30 p.m.	1-1:30 p.m.	1-1:30 p.m.	1-1:30 p.m.	1-1:30 p.m.
1:30-2 p.m.	1:30-2 p.m.	1:30-2 p.m.	1:30-2 p.m.	1:30-2 p.m.	1:30-2 p.m.	1:30-2 p.m.	1:30-2 p.m.
2-2:30 p.m.	2-2:30 p.m.	2-2:30 p.m.	2-2:30 p.m.	2-2:30 p.m.	2-2:30 p.m.	2-2:30 p.m.	2-2:30 p.m.
2:30-3 p.m.	2:30-3 p.m.	2:30-3 p.m.	2:30-3 p.m.	2:30-3 p.m.	2:30-3 p.m.	2:30-3 p.m.	2:30-3 p.m.
3-3:30 p.m.	3-3:30 p.m.	3-3:30 p.m.	3-3:30 p.m.	3-3:30 p.m.	3-3:30 p.m.	3-3:30 p.m.	3-3:30 p.m.
3:30-4 p.m.	3:30-4 p.m.	3:30-4 p.m.	3:30-4 p.m.	3:30-4 p.m.	3:30-4 p.m.	3:30-4 p.m.	3:30-4 p.m.
4-4:30 p.m.	4-4:30 p.m.	4-4:30 p.m.	4-4:30 p.m.	4-4:30 p.m.	4-4:30 p.m.	4-4:30 p.m.	4-4:30 p.m.
4:30-5 p.m.	4:30-5 p.m.	4:30-5 p.m.	4:30-5 p.m.	4:30-5 p.m.	4:30-5 p.m.	4:30-5 p.m.	4:30-5 p.m.
5-5:30 p.m.	5-5:30 p.m.	5-5:30 p.m.	5-5:30 p.m.	5-5:30 p.m.	5-5:30 p.m.	5-5:30 p.m.	5-5:30 p.m.
5:30-6 p.m.	5:30-6 p.m.	5:30-6 p.m.	5:30-6 p.m.	5:30-6 p.m.	5:30-6 p.m.	5:30-6 p.m.	5:30-6 p.m.
6-6:30 p.m.	6-6:30 p.m.	6-6:30 p.m.	6-6:30 p.m.	6-6:30 p.m.	6-6:30 p.m.	6-6:30 p.m.	6-6:30 p.m.
6:30-7 p.m.	6:30-7 p.m.	6:30-7 p.m.	6:30-7 p.m.	6:30-7 p.m.	6:30-7 p.m.	6:30-7 p.m.	6:30-7 p.m.
7-7:30 p.m.	7-7:30 p.m.	7-7:30 p.m.	7-7:30 p.m.	7-7:30 p.m.	7-7:30 p.m.	7-7:30 p.m.	7-7:30 p.m.
7:30-8 p.m.	7:30-8 p.m.	7:30-8 p.m.	7:30-8 p.m.	7:30-8 p.m.	7:30-8 p.m.	7:30-8 p.m.	7:30-8 p.m.
8-8:30 p.m.	8-8:30 p.m.	8-8:30 p.m.	8-8:30 p.m.	8-8:30 p.m.	8-8:30 p.m.	8-8:30 p.m.	8-8:30 p.m.
8:30-9 p.m.	8:30-9 p.m.	8:30-9 p.m.	8:30-9 p.m.	8:30-9 p.m.	8:30-9 p.m.	8:30-9 p.m.	8:30-9 p.m.
9-9:30 p.m.	9-9:30 p.m.	9-9:30 p.m.	9-9:30 p.m.	9-9:30 p.m.	9-9:30 p.m.	9-9:30 p.m.	9-9:30 p.m.
9:30-10 p.m.	9:30-10 p.m.	9:30-10 p.m.	9:30-10 p.m.	9:30-10 p.m.	9:30-10 p.m.	9:30-10 p.m.	9:30-10 p.m.
10-10:30 p.m.	10-10:30 p.m.	10-10:30 p.m.	10-10:30 p.m.	10-10:30 p.m.	10-10:30 p.m.	10-10:30 p.m.	10-10:30 p.m.
10:30-11 p.m.	10:30-11 p.m.	10:30-11 p.m.	10:30-11 p.m.	10:30-11 p.m.	10:30-11 p.m.	10:30-11 p.m.	10:30-11 p.m.
11-11:30 p.m.	11-11:30 p.m.	11-11:30 p.m.	11-11:30 p.m.	11-11:30 p.m.	11-11:30 p.m.	11-11:30 p.m.	11-11:30 p.m.
11:30-12 a.m.	11:30-12 a.m.	11:30-12 a.m.	11:30-12 a.m.	11:30-12 a.m.	11:30-12 a.m.	11:30-12 a.m.	11:30-12 a.m.

CODE NUMBER: __________

14. I prefer to sleep in a completely dark room: ___yes ___no.

15. I wake up more easily when morning light shines into my room: ___yes ___no.

16. On days off when I wake up I generally need____ minutes to get out of bed after I've woken up.

17. Have you noticed any difference in the regularity of your menstrual cycle while working night vs. day shifts? (if relevant to your work experience and age): ___yes___no

18. If you take naps when you have a chance to, on which day(s) of the week do you tend to take naps. (please answer in terms of ABCDEFG from the sample work week above, and indicate these nap times on the preceding chart[s].)

    Day A____ Day B____ Day C____ Day D____ Day E____ Day F____ Day G____

    Please review the chart(s) you completed on the previous pages to make sure you precisely indicated all regular sleeping times, **including** naps.

19. On average, how much time do you spend outside, exposed to sunlight?
    On work days: ___hours ___minutes
    On free days: ___hours ___minutes

PLEASE PROCEED TO THE FINAL PAGES

CODE NUMBER: __________

Self assessment

After you have answered the preceding questions, you should have an idea about which "time of day" type you are. If for example, you like (and manage) to sleep quite a bit longer on free days than on workdays, or you cannot get out of bed on Monday mornings, even without a Sunday-night party, then you are more of a "late" type. If, however, you regularly wake up and feel perky once you jump out of bed, and if you would rather go to bed early than participate in an evening activity, then you are an "early" type. In the following questions, please categorize yourself and your family members. If you work the night shift, please answer this question for yourself from your experience living on an ordinary schedule (i.e., not a night shift schedule). Mark only one possibility for each line!

Description of categories:

- extreme early type = 0
- moderate early type = 1
- slight early type = 2
- "normal" type = 3
- slight late type = 4
- moderate late type = 5
- extreme late type = 6

**I am** …	0 ☐	1 ☐	2 ☐	3 ☐	4 ☐	5 ☐	6 ☐
**as a child, I was** …	0 ☐	1 ☐	2 ☐	3 ☐	4 ☐	5 ☐	6 ☐
**as teenager, I was**…	0 ☐	1 ☐	2 ☐	3 ☐	4 ☐	5 ☐	6 ☐
**In case you are older than 65:** in the middle of my life, I was …	0 ☐	1 ☐	2 ☐	3 ☐	4 ☐	5 ☐	6 ☐
My parents are/were… **Mother** …	0 ☐	1 ☐	2 ☐	3 ☐	4 ☐	5 ☐	6 ☐
**Father** …	0 ☐	1 ☐	2 ☐	3 ☐	4 ☐	5 ☐	6 ☐
**My siblings are/were … (please underline Brother or Sister)**							
Brother/Sister	0 ☐	1 ☐	2 ☐	3 ☐	4 ☐	5 ☐	6 ☐
Brother/Sister	0 ☐	1 ☐	2 ☐	3 ☐	4 ☐	5 ☐	6 ☐
Brother/Sister	0 ☐	1 ☐	2 ☐	3 ☐	4 ☐	5 ☐	6 ☐
Brother/Sister	0 ☐	1 ☐	2 ☐	3 ☐	4 ☐	5 ☐	6 ☐
Brother/Sister	0 ☐	1 ☐	2 ☐	3 ☐	4 ☐	5 ☐	6 ☐
Brother/Sister	0 ☐	1 ☐	2 ☐	3 ☐	4 ☐	5 ☐	6 ☐
Brother/Sister	0 ☐	1 ☐	2 ☐	3 ☐	4 ☐	5 ☐	6 ☐
**My partner, if applicable** (girl/boyfriend, spouse, significant other) **is/was …**	0 ☐	1 ☐	2 ☐	3 ☐	4 ☐	5 ☐	6 ☐

CODE NUMBER __________

Please complete the following demographic information:

Gender: ___male ___female

Age: ___ years

How many children are living in your household?
___none ___1 – 3 ___4 – 6 ___more than 6

If there are children in the home, what are their age ranges (check all that apply):
___0 – 2 years ___3 – 5 years ___6 – 10 years ___older than 10 years

Thank you for taking the time to fill out this questionnaire and contribute to our research. We appreciate your help.

Revised 10/03/05
