# Supplementary material for: Shift Work in Nurses: Contribution of Phenotypes and Genotypes to Adaptation
Source: PLoS One. 2011 Apr 13;6(4):e18395. doi: 10.1371/journal.pone.0018395 (PMC3076422; doi:10.1371/journal.pone.0018395)
Supplement: Table S1 — Shift types represented in the sample. (DOC) [file pone.0018395.s002.doc]

**Supporting Information**

Table S1. Shift Types Represented in the Sample.

| Shift | N |
| --- | --- |
| 12-hour Day | 107 |
| 12-hour Night | 212 |
| 8-hour Day | 14 |
| 8-hour Evening | 4 |
| 8-hour Night | 4 |
| 12-hour Day (other schedule) | 6 |
| 12-hour Night (other schedule) | 0 |
| 8-hour Day (other schedule) | 8 |
| 8-hour Night (other schedule) | 0 |
| Other Shift | 26 |
| Office Hours | 32 |

Note: Nurses had the option to complete the schedule for more than one shift (e.g., if they have experienced working on more than one shift), and therefore, the total number of current shift types is greater than the total N for the study.
