# Supplementary material for: Shift Work in Nurses: Contribution of Phenotypes and Genotypes to Adaptation
Source: PLoS One. 2011 Apr 13;6(4):e18395. doi: 10.1371/journal.pone.0018395 (PMC3076422; doi:10.1371/journal.pone.0018395)
Supplement: Table S2 — Genic location and minor allele frequencies (MAF) of candidate gene polymorphisms. (DOC) [file pone.0018395.s003.doc]

**Supporting Information**

Table S2. Genic location and minor allele frequencies (MAF) of candidate gene polymorphisms.

| Polymorphism | rs numbers | MAF | Genic Location | Base change | Amino acid sequence change | Reference |
| --- | --- | --- | --- | --- | --- | --- |
| ARNTL1.1 | rs70965441 | 0.0304 | 5'UTR | A>C | n/a | [6] |
| ARNTL1.2 | rs70965440 | 0.0084 | intron 1 | T>G ** | n/a | [6] |
| ARNTL1.3 | rs70965442 | 0.0097 | intron 5 | G>A ** | n/a | [6] |
| ARNTL2.1 | rs5797225 | 0.2921 | promoter | -/TTG | n/a | [6] |
| ARNTL2.2 | rs7137588 | 0.2744 | promoter | G>C** | n/a | [6] |
| ARNTL2.3 | rs11048972 | 0.0639 | promoter | A>G | n/a | [6] |
| ARNTL2.4 | rs70965443 | 0.0401 | promoter | A>G | n/a | [6] |
| ARNTL2.5 | rs10548381 | 0.0968 | promoter | CTA/-** | n/a | [6] |
| ARNTL2.6 | rs4964059 | 0.3317 | intron 3 | A>C | n/a | [6] |
| ARNTL2.7 | rs70965445 | 0.0011 | 3'flanking | A>G | n/a | [6] |
| CLOCK.1 | rs70965446 | 0.0024 | 3'UTR | A>G | n/a | [6] |
| CLOCK.2 | rs1801260 | 0.1065 | 3'UTR | T>C** | n/a | [7,8,9,12,13] |
| NPAS2.1 | rs1811399 | 0.2399 | intron 1 | T>G** | n/a | [15] |
| NPAS2.2 | rs2117714 | 0.2832 | intron 2 | T>C** | n/a | [15] |
| NPAS2.3 | rs4851377 | 0.4622 | intron 2 | T>C** | n/a | [16,17] |
| NPAS2.4 | rs34705978 | 0.1675 | intron 3 | C>T | n/a | [15] |
| NPAS2.5 | rs17717414 | 0.1929 | intron 3 | A>G | n/a | [16,17] |
| PER2.1 | rs2304669 | 0.1295 | exon 17 | A>G | syn | [6] |
| PER2.2 | FASPS | 0.0012 | exon 17 | A>G | S>G | [24] |
| PER2.3 | rs70965448 | 0.0051 | exon 17 | C>T | A>V |  |
| PER2.4 | rs2304670 | 0.0733 | exon 17 | G>A** | syn | [6] |
| PER2.5 | rs2304671 | 0.0721 | exon 17 | G>A** | syn | [6] |
| PER3.1 | rs228669 | 0.0303 | exon 11 | T>C | syn | [18] |
| PER3.2 | NA | 0.0024 | exon 17 | A>G | Syn | [6] |
| PER3.3 | rs228696 | 0.0266 | exon 17 | T>C** | L>P | [6] |
| PER3.4 | rs228697 | 0.0877 | exon 17 | C>G** | P>A | [6] |
| PER3.5 | rs17031614 | 0.0316 | exon 17 | G>A** | syn | [6] |
| PER3.6 | VNTR | 0.3425 | exon 18 | 54bp VNTR , (4-5 copies) | -18aa | [18,19,20,21,22,23] |
| PER3.7 | rs10462021 | 0.1743 | exon 20 | A>G | H>R | [6] |
| AA-NAT.1 | rs4238989 | 0.4935 | 5' flanking | C>G | n/a | [14] |
| AA-NAT.2 | rs3760138 | 0.4973 | 5' flanking | G>T | n/a | [14] |
| MTNR1A.1 | rs8192550 | 0.0309 | exon 2 | A>G | Syn | C.H. Johnson lab |
| MTNR1B.1 | rs10830963 | 0.2876 | intron 1 | C>G | n/a | [10,11] |
| MTNR1B.2 | rs1562444 | 0.4786 | 3’UTR | A>G | n/a | C.H. Johnson lab |
| MTNR1B.3 | rs12792653 | 0.4006 | 3’UTR | A>G | n/a | C.H. Johnson lab |
