# Supplementary material for: Shift Work in Nurses: Contribution of Phenotypes and Genotypes to Adaptation
Source: PLoS One. 2011 Apr 13;6(4):e18395. doi: 10.1371/journal.pone.0018395 (PMC3076422; doi:10.1371/journal.pone.0018395)
Supplement: Methods S1 — (DOC) [file pone.0018395.s004.doc]

**Supporting Information**

**Supplementary Methods**

**Genotyping**

*Marker Selection:* The mammalian biological clock is composed of autoregulatory transcription/translation feedback loops in the expression of central clock genes, including *Arntl/Bmal1, Arntl2/Bmal2, Clock, Npas2, Per1, Per2*, and *Per3* [1]. In addition, the clock-controlled hormone melatonin also plays an important role in circadian and sleep systems through its receptors encoded by the *MTNR* genes and its synthetic pathway that includes *AA-NAT* [2]. The specific clock gene variants selected for this study were based on being common polymorphisms in a candidate gene approach. We consider the study of common polymorphisms to be of more potential explanatory value for understanding phenotypes in human populations than the study of rare polymorphisms. For the SNPs in the *Arntl* (OMIM 126110, aka *Bmal1* or *Mop3*) [3,4] and *Arntl2* (OMIM 602550, aka *Bmal2* or *Mop9*) [5] genes, we performed systematic screening and characterization of common exonic and promoter polymorphisms [6] and selected the common polymorphisms in this particular population of European-American nurses. For *Clock, Npas2, Per1, Per2*, and *Per3*, we chose previously reported polymorphisms that were relatively common and that had been associated with a host of disorders [6,7,8,9,10,11,12,13,14,15,16,17,18,19,20,21,22,23,24] and included a few other SNPs in these genes that we discovered by serendipity.

*DNA Isolation and Primer Design:* Briefly, 5 cc whole blood was collected from each subject and DNA was isolated from lymphocytes by the Vanderbilt DNA core facility. Previously reported primers were designed and used for the ARNTL, ARNTL2, AA-NAT, PER2, PER3, CLOCK and NPAS2 variants as previously described [6].

*Sequenom screening:* The following SNPs were analyzed by Sequenom (Sequenom Inc. San Diego Ca): *MTNR1A*: rs8192550, *MTNR1B*: rs10830963, rs1562444, rs12792653, *PER2*: rs70965448, *PER3*: rs228697, rs17031614, *NPAS2*: rs1811399, rs2117714, rs4851377, rs34705978, rs17717414, *AANAT*: rs4238989, rs3760138. The samples were loaded in 384-well plates and genotyped by PCR primer extension assay and the single base extension products were detected by Mass ARRAY MALDI-TOF mass spectrometer with a Sequenom iPLEX genotyping platform. The sequence difference at the single nucleotide level was detected as an allele-specific difference in mass between extension products. The difference in mass was correlated to a specific genotype. The call rate threshold for all the SNPs analyzed by Sequenom was at least 95%.

*Single-stranded conformation polymorphism (SSCP) screening and genotyping.* SNPs that were rejected by the software during Sequenom assay design were run in SSCP gels; these SNPS were: *PER2*: rs2304669, FASPS, rs2304670, rs2304671; *PER3* rs228669, rs228696, rs10462021; *CLOCK* rs70965446, rs1801260; *ARNTL*: rs70965440, rs70965441, rs70965442 and *ARNTL2* rs7137588, rs10548381, rs4964059, rs70965445, rs11048972, rs70965443, rs5797225. Each amplicon was PCR-amplified from the genomic DNA using one of two protocols: Platinum PCR Supermix (Invitrogen, Carlsbad, CA, USA) was used for *AANAT,* *PER2*, *PER3,* *CLOCK and NPAS2*. PCR for these genes was run with the following parameters: 95°C for 1.5 min, then 35 cycles of 95°C for 30 sec, 55°C for 30 sec and 72°C for 30 sec, followed by a final extension at 72°C for 7 min and a 4°C hold. For *ARNTL* and *ARNTL2*, AmpliTaq Gold (Applied Biosystems, Foster City, CA, USA) was used with the provided PCR Buffer II and MgCl_2_. PCR for *ARNTL* and *ARNTL2* was run with the following parameters: 95°C for 7.5 min, then 35 cycles of 95°C for 30 sec, 55°C for 30 sec and 72°C for 30 sec, followed by a final extension at 72°C for 15 min and a 4°C hold. Loading dye (4X = 40 mM EDTA, 0.1% bromophenol blue, 0.1% xylene cyanol prepared in deionized formamide) was added to all PCR products. These products were then denatured at 94°C for 3 min, rapidly cooled to 4°C, and separated in MDE electrophoresis gels (Lonza, Basel, Switzerland) at 15 W in 0.6x TBE running buffer at 4°C for 8–14 h, depending on the size and characteristics of the amplimer. Gels were developed by silver staining to visualize bands [25]. Novel common polymorphisms were identified in a previous report [6]. All variants were quantified and tabulated within a sample-specific spreadsheet. Finally, SNP allele frequencies in all genes were determined with SSCP on all the samples using the SSCP patterns as confirmed by sequencing. In the case of the variable nucleotide tandem repeat in *PER3* (AB047536), SSCP was not necessary; rather, the PCR products were simply separated by electrophoresis in a 2% agarose gel and visualized by ethidium bromide staining. This method easily distinguished between the 5-repeat allele (414bp) and the 4-repeat allele (360bp).

*Sequenom Sequence Analysis*

Amplicons with band-shifts were PCR amplified as stated above, cleaned with QIAQuick PCR Purification Kit (QIAGEN, Valencia, CA), and sequenced with an ABI 3730xl DNA Analyzer (Applied Biosystems). Control and shifted sequences were aligned using Clustal W, and variants were compared to dbSNP build 126 for Homo sapiens (NCBI, Bethesda, MD, USA).

**Statistical Analyses**

SPSS 13.0 was used for the descriptive statistics and analyses of the outcome variables from the survey. In general, categorical data were analyzed with a contingency analysis, using Pearson’s Chi-square test or the likelihood ration (G). Correlations between scaled variables were determined by Pearson’s R. For differences among normally distributed groups with homogenous variances (determined by Kolmogorov-Smirnov and Levene’s tests, respectively), we used independent samples t-tests (for two groups) and univariate analyses of variance (ANOVA; for three or more groups). For samples with a significant Levene’s test, either Mann-Whitney U (for two groups) or Kruskal Wallis (for three or more groups) tests were used. In the case of an interaction, a Scheirer-Ray-Hare extension of the Kruskal Wallis test was used. For the multi-variate analysis of the effects of strategy types, caffeine consumption was normalized by the median age (36) since age was significantly different among the strategy types (F(4,286) = 4.52, *p* < 0.01, Table S2), and caffeine consumption was significantly predicted by age (R^2^ = 0.072, *p* < 0.01). Therefore, the dependent variable Caffeine was adjusted by the following equation, based on the significant parameters in the linear regression between age and caffeine: Age-adjusted caffeine = Caffeine - ((Age - 36) * .054) + 1.056. In an effort to provide a more descriptive analysis of the phenotype data, multiple tests were conducted on the entire dataset and multiple comparisons were not controlled except at the univariate level.

A total of 35 polymorphisms, three in *ARNTL*, seven in *ARNTL2*, five in *NPAS2*, two in *CLOCK*, five in *PER2*, seven in *PER3*, two in *AA-NAT*, one in *MTNR1A*, and three in *MTNR1B* (see Table S2), were analyzed for association with the following outcome variables: “average weekly alcohol intake” (Figure S1, #12), “daily caffeine consumption” (Figure S1, #11), “likelihood to doze” (Figure S1, #10), “self-assessed chronotype” (Figure S1, page 7), “minutes to get out of bed” (Figure S1, #16), as well as variables associated with/obtained from the typical schedule chart: “work-day sleep duration,” “work-day midsleep time,” “free-day sleep duration, “free-day midsleep time” (unadjusted and MSF_SC_), “sleep sessions,” “off-shift sleep phase (corrected for sleep debt),” “sleep strategy,” “total sleep duration,” and “adaptation” as described above. For each outcome variable analyzed, significant covariates (most commonly, “age” and “children at home”) were selected with backwards step-wise variable selection (with *p* < 0.05 as the criteria for model inclusion and exclusion) for inclusion as covariate(s) in the association tests. After careful quality control (based on genotyping efficiency >90% for both individuals and polymorphisms), and tests for deviation from Hardy-Weinberg Equilibrium as previously described [6], polymorphisms were evaluated for potential phenotype associations. For each polymorphism and for each outcome, potential genotype-phenotype associations were tested using logistic regression, ordinal/trend analysis, and regression for binary, ordinal, and continuous outcomes, respectively, after controlling for the covariates selected from backwards selection. For the association analysis, dummy variables representing the three possible genotypes at each polymorphism were tested, such that no genetic model assumptions were made. To control for multiple comparisons, 1000 permutations (permuting the polymorphism values in the data) were used to account for tests across polymorphisms and across outcomes to determine an empirical p-value cut-off that corresponds to a family-wise type I error rate of 5%. Therefore, raw p-values for individual tests of association were considered significant if they were less than 0.009. For outcome variables derived from the typical schedule part of the survey [i.e., “work-day sleep duration,” “work-day midsleep time,” “free-day sleep duration, “free-day midsleep time” (unadjusted and MSF_SC_), “sleep sessions,” “off-shift sleep phase (corrected for sleep debt),” “sleep strategy,” “total sleep duration,” and “adaptation”], some subjects had completed schedules for both day and night-shift. To avoid duplicate genetic data for these subjects, the entire dataset was analyzed two ways: first, with all shifts included together (unstratified) and second, stratified for shift-type during the analysis of these variables. For the unstratified approach, care was taken to remove duplicate entries for nurses who included sleep schedule tables for both day- and night-shifts. The strength of this stratification approach is that it allowed comparison of associations during different shift environments; however, the weakness of this strategy is that the overall sample size was decreased, resulting in a loss of power. Therefore, it is likely that some associations may not have been detected in our analysis. For the directed hypothesis test of Genotype X Strategy interactions on self-reported adaptation, we performed univariate analyses (two-way ANOVA) for each polymorphism for the two most common strategies (Switch Sleepers and No Sleep); due to the decreased sample size after limiting the analysis to the strategy types, these tests were not corrected for multiple testing and thus, these results should be replicated before generalized.

We used the Generalized Multifactor Dimensionality Reduction (GMDR) method [26] to evaluate potential multi-locus interactions that predict each phenotype (after adjusting for significant covariates). GMDR was developed as an extension of the Multifactor Dimensionality Reduction (MDR) algorithm to detect gene-gene and gene-environment interactions in data with quantitative or discrete input and output variables. GMDR introduces the concept of a score statistic into the MDR framework to obtain an appropriate statistic to classify multifactor contingency table cells into two different groups. The score value is based on a generalized linear model of the phenotype variable, and allows for the use of covariates in the score approximation. Details of the score statistic are found in Lou et al. [26]. Briefly, the steps of GMDR are as follows. In step one, data was divided into a training set and an independent testing set for cross validation. Five-fold cross-validation was used, with 4/5 of the data used for training and 1/5 for testing. A set of n genetic factors were then selected. These factors and their multiple classes were divided in n-dimensional space. In the traditional MDR approach, the ratios of cases to controls were then calculated within each multifactor class. Each multifactor cell class was labeled “high risk” if the ratio exceeded 1.0, or “low risk” if less than 1.0, thus reducing n-dimensional space to one dimension with two levels. In GMDR, the ratio of cases to controls in each cell is replaced by the score values. The null hypothesis assumes there are no effects of the putative factors or their interactions, so the score values are the same for all different factor classifications. In the third step of GMDR, the cumulative score value was calculated within each multifactor cell and in the fourth step, each multifactor cell was labeled either as high-risk if the average score meets or exceeded a pre-assigned threshold T (e.g., 0), or as low-risk if the threshold was not exceeded. The collection of these multifactor classes comprised the GMDR model. For each possible model size (one-locus, two-locus… n-locus) the single GMDR model that misclassified the fewest individuals was chosen. Again, the score values for the number of cases and controls are used to evaluate the errors. Prediction error is then calculated using 5-fold cross-validation. The result is a set of models, one for each model size considered. The final model was chosen that minimizes prediction error while maximizing cross validation consistency (CVC). The statistical significance of the final best model was determined through permutation testing, which involved creating 1000 permuted datasets by randomizing the value of the phenotype variable. The entire procedure was repeated for each, generating a distribution of 1000 prediction errors that could be expected by chance alone. The significance of the final model was determined by comparing the prediction error of the final model to the distribution. A p-value was extracted for the model by its theoretical location in the permutation distribution. In the present study, GMDR analysis was performed with the same outcome variables listed above as the phenotypes, using stratification when necessary and all other genetic markers as potential predictor variables. Analysis was performed with 5-fold cross-validation, and single-variable through four-variable interactions were evaluated. Analyses were performed using the Java-based GUI freely available from <http://www.healthsystem.virginia.edu/internet/addiction-genomics/software/gmdr.cfm> [26]. The demographic covariates identified for each phenotype by the backwards selection described above, and the identified covariate were used as covariates in the GMDR analysis. Again, permutation testing was used to empirically define the prediction accuracies that are significant with a family-wise type I error rate of 0.05.

**Supplementary Material References**

1. Dunlap JC, Loros JJ, DeCoursey PJ (2004) Chronobiology : biological timekeeping. Sunderland, Mass.: Sinauer Associates. xix, 406 p. p.

2. Lewy AJ, Lefler BJ, Emens JS, Bauer VK (2006) The circadian basis of winter depression. Proc Natl Acad Sci U S A 103: 7414-7419.

3. Hogenesch JB, Gu YZ, Jain S, Bradfield CA (1998) The basic-helix-loop-helix-PAS orphan MOP3 forms transcriptionally active complexes with circadian and hypoxia factors. Proc Natl Acad Sci U S A 95: 5474-5479.

4. Takahata S, Sogawa K, Kobayashi A, Ema M, Mimura J, et al. (1998) Transcriptionally active heterodimer formation of an Arnt-like PAS protein, Arnt3, with HIF-1a, HLF, and clock. Biochem Biophys Res Commun 248: 789-794.

5. Hogenesch JB, Gu YZ, Moran SM, Shimomura K, Radcliffe LA, et al. (2000) The basic helix-loop-helix-PAS protein MOP9 is a brain-specific heterodimeric partner of circadian and hypoxia factors. J Neurosci 20: RC83.

6. Ciarleglio CM, Ryckman KK, Servick SV, Hida A, Robbins S, et al. (2008) Genetic differences in human circadian clock genes among worldwide populations. J Biol Rhythms 23: 330-340.

7. Takao T, Tachikawa H, Kawanishi Y, Mizukami K, Asada T (2007) CLOCK gene T3111C polymorphism is associated with Japanese schizophrenics: a preliminary study. Eur Neuropsychopharmacol 17: 273-276.

8. Iwase T, Kajimura N, Uchiyama M, Ebisawa T, Yoshimura K, et al. (2002) Mutation screening of the human Clock gene in circadian rhythm sleep disorders. Psychiatry Res 109: 121-128.

9. Serretti A, Cusin C, Benedetti F, Mandelli L, Pirovano A, et al. (2005) Insomnia improvement during antidepressant treatment and CLOCK gene polymorphism. Am J Med Genet B Neuropsychiatr Genet 137B: 36-39.

10. Lyssenko V, Nagorny CL, Erdos MR, Wierup N, Jonsson A, et al. (2009) Common variant in MTNR1B associated with increased risk of type 2 diabetes and impaired early insulin secretion. Nat Genet 41: 82-88.

11. Prokopenko I, Langenberg C, Florez JC, Saxena R, Soranzo N, et al. (2009) Variants in MTNR1B influence fasting glucose levels. Nat Genet 41: 77-81.

12. Katzenberg D, Young T, Finn L, Lin L, King DP, et al. (1998) A CLOCK polymorphism associated with human diurnal preference. Sleep 21: 569-576.

13. Robilliard DL, Archer SN, Arendt J, Lockley SW, Hack LM, et al. (2002) The 3111 Clock gene polymorphism is not associated with sleep and circadian rhythmicity in phenotypically characterized human subjects. J Sleep Res 11: 305-312.

14. Wang GY, Lee CG, Lee EJ (2004) Genetic variability of arylalkylamine-N-acetyl-transferase (AA-NAT) gene and human sleep/wake pattern. Chronobiol Int 21: 229-237.

15. Nicholas B, Rudrasingham V, Nash S, Kirov G, Owen MJ, et al. (2007) Association of Per1 and Npas2 with autistic disorder: support for the clock genes/social timing hypothesis. Mol Psychiatry 12: 581-592.

16. Partonen T, Treutlein J, Alpman A, Frank J, Johansson C, et al. (2007) Three circadian clock genes Per2, Arntl, and Npas2 contribute to winter depression. Ann Med 39: 229-238.

17. Englund A, Kovanen L, Saarikoski ST, Haukka J, Reunanen A, et al. (2009) NPAS2 and PER2 are linked to risk factors of the metabolic syndrome. J Circadian Rhythms 7: 5.

18. Ebisawa T, Uchiyama M, Kajimura N, Mishima K, Kamei Y, et al. (2001) Association of structural polymorphisms in the human period3 gene with delayed sleep phase syndrome. EMBO Rep 2: 342-346.

19. Archer SN, Robilliard DL, Skene DJ, Smits M, Williams A, et al. (2003) A length polymorphism in the circadian clock gene Per3 is linked to delayed sleep phase syndrome and extreme diurnal preference. Sleep 26: 413-415.

20. Pereira DS, Tufik S, Louzada FM, Benedito-Silva AA, Lopez AR, et al. (2005) Association of the length polymorphism in the human Per3 gene with the delayed sleep-phase syndrome: does latitude have an influence upon it? Sleep 28: 29-32.

21. Viola AU, Archer SN, James LM, Groeger JA, Lo JC, et al. (2007) PER3 polymorphism predicts sleep structure and waking performance. Curr Biol 17: 613-618.

22. Jones KH, Ellis J, von Schantz M, Skene DJ, Dijk DJ, et al. (2007) Age-related change in the association between a polymorphism in the PER3 gene and preferred timing of sleep and waking activities. J Sleep Res 16: 12-16.

23. Nadkarni NA, Weale ME, von Schantz M, Thomas MG (2005) Evolution of a length polymorphism in the human PER3 gene, a component of the circadian system. J Biol Rhythms 20: 490-499.

24. Toh KL, Jones CR, He Y, Eide EJ, Hinz WA, et al. (2001) An hPer2 phosphorylation site mutation in familial advanced sleep phase syndrome. Science 291: 1040-1043.

25. Nataraj AJ, Olivos-Glander I, Kusukawa N, Highsmith WE, Jr. (1999) Single-strand conformation polymorphism and heteroduplex analysis for gel-based mutation detection. Electrophoresis 20: 1177-1185.

26. Lou XY, Chen GB, Yan L, Ma JZ, Zhu J, et al. (2007) A generalized combinatorial approach for detecting gene-by-gene and gene-by-environment interactions with application to nicotine dependence. Am J Hum Genet 80: 1125-1137.

27. Roenneberg T, Wirz-Justice A, Merrow M (2003) Life between clocks: daily temporal patterns of human chronotypes. J Biol Rhythms 18: 80-90.
